# bifidoAnnotator: fine-grained annotation of bifidobacterial glycoside hydrolases for human milk glycan utilization

**DOI:** 10.1099/mgen.0.001702

**Published:** 2026-04-27

**Authors:** Nicholas Pucci, Daniel R. Mende

**Affiliations:** 1 Department of Medical Microbiology & Infectious Diseases, Amsterdam UMC, Location AMC at University of Amsterdam, 1105 AZ, Amsterdam, The Netherlands; 2 Human Biology-Microbiome-Quantum Research Center (WPI-Bio2Q), Keio University, 108-8345, Tokyo, Japan

**Keywords:** bifidobacteria, bioinformatics, genome annotation, glycoside hydrolase (GH), human milk glycan (HMG) metabolism, microbiome

## Abstract

Human milk glycan (HMG) metabolism, especially by bifidobacteria, is crucial for infant gut colonization and healthy microbiome development. Bifidobacterial species and even strains are highly variable in their ability and in their enzymatic repertoire for HMG metabolism. The enzymes involved in HMG metabolism often have many non-HMG-related homologues, necessitating fine-grained annotation for accurate assessment of bifidobacterial HMG metabolic capabilities. However, current annotation tools provide only broad glycoside hydrolase (GH) (sub)family classifications. Here, we present *bifidoAnnotator*, a tool for fine-grained annotation and visualization of bifidobacterial GH genes involved in HMG utilization. *bifidoAnnotator* leverages MMseqs2 (Many-against-Many sequence searching) to map protein sequences against a manually curated database of over 22,000 bifidobacterial GH proteins, organized into 13 families and 108 functional clusters, each assigned a validation status (i.e. experimentally validated, putative or hypothetical). The tool performs hierarchical annotation at family and cluster levels, identifying consistently annotated protein variants rather than just broad family assignments, and generates publication-ready heatmaps for comparative analysis. Benchmarking on a gold standard dataset demonstrated that bifidoAnnotator has superior performance (95.9% precision, 100% recall) compared with six established tools and is an order of magnitude faster than the most accurate competitor. *bifidoAnnotator*’s superior performance and computational efficiency represent a meaningful advance in high-throughput genomic annotation workflows, enabling detailed characterization of strain-level functional diversity in bifidobacterial HMG metabolism.

**Impact Statement**

Understanding strain-specific human milk glycan (HMG) metabolic capabilities is essential for characterizing bifidobacterial function in infant gut development and probiotic selection. However, current annotation tools provide only broad glycoside hydrolase (GH) family classifications that fail to resolve functionally distinct enzyme variants. Many GH families contain both HMG-specific and non-HMG-related homologues with distinct substrate specificities, preventing accurate assessment of strain-specific breastmilk glycan utilization. Here, we present bifidoAnnotator, a tool for detailed annotation and visualization of bifidobacterial GH genes involved in HMG utilization. *bifidoAnnotator* leverages a manually curated database of over 22,000 bifidobacterial GH proteins from 13 families, organized into 108 functional clusters with experimental validation status. Using MMseqs2 (Many-against-Many sequence searching), bifidoAnnotator performs hierarchical annotation, assigning proteins to GH families and functional clusters, distinguishing enzyme variants with different substrate preferences that are masked by traditional classifications. Benchmarking against nine genomes with experimentally validated repertoires demonstrated superior precision (95.9%) compared with established tools (database for automated Carbohydrate-active enzyme ANnotation, dbCAN; enzyme Classification And Motif Identification, eCAMI; Conserved Unique Peptide Patterns, CUPP) while maintaining 100% recall. Application to 49 bifidobacterial metagenome-assembled genomes from a mother–infant cohort successfully revealed functional specializations, including extracellular vs. intracellular sialidases and strain-level variation in *α*-mannosidases and endo-*β*-*N*-acetylglucosaminidases. *bifidoAnnotator*’s high, functionally relevant resolution enables GH enzymatic repertoires associated with HMG degradation at scale, supporting comparative genomics, functional microbiome profiling, investigation of bifidobacterial ecological dynamics and evidence-based probiotic selection.

## Data Summary

The *bifidoAnnotator*’s source code, documentation and bifidobacterial genomes used for benchmarking are available at https://github.com/nicholaspucci/bifidoAnnotator. The bifidoAnnotator database (*bifDB*) and Amsterdam Infant Microbiome Study (AIMS) bifidobacterial metagenome-assembled genomes are deposited in Zenodo (DOI 10.5281/zenodo.19133752). *bifidoAnnotator* is installable via conda.

## Introduction

Bifidobacteria are among the first colonizers of the human gastrointestinal microbiome, playing a pivotal role in promoting infant health and development [1,4]. These bacteria utilize indigestible human milk glycans (HMGs) as their main energy source: human milk oligosaccharides (HMOs) and *N*-linked glycans. Several infant-associated species, including *Bifidobacterium breve*, *Bifidobacterium bifidum* and *Bifidobacterium longum* (subsp. *infantis* and subsp. *longum*), are particularly efficient HMG degraders [5,8], utilizing diverse HMG transporters and degrading enzymes. The diversity and copy number of HMG-degrading enzymes vary significantly among bifidobacterial species and strains, directly influencing their carbohydrate-degrading capacity and ecological fitness in the infant gut, with potential repercussions for gut microbiome development [6,8,10]. Understanding strain-specific HMG-degrading enzyme repertoires has proven essential for predicting functional differences between bifidobacteria [11,12] and guiding rational probiotic selection [13,14].

To characterize these HMG-degrading enzymes, researchers often use general carbohydrate-acting enzyme (CAZy) databases (e.g. CAZy db [15]), which provide annotations of glycoside hydrolases (GHs) at the family level. While family-level annotations provide a general overview of HMG utilization potential, they lack the resolution necessary to understand specific bifidobacterial HMG utilization [16,20]. For example, GH2-family enzymes include beta-galactosidases, as well as beta-mannosidases and beta-glucuronidases [21]. Furthermore, GH2 beta-galactosidases display distinct substrate specificities, with some being active on lacto-*N*-neotetraose (LNnT) and others on lactose [22,23]. Similar observations have also been made for other GH families. Similarly, while some tools offer subfamily-level resolution (e.g. enzyme Classification And Motif Identification, eCAMI [24]), designed to group enzymes with similar biochemical activities, they may still group functionally distinct HMG-utilizing variants together [24,25]. Another approach is the reconstruction of full HMG degradation pathways as in *glycobif* [26], yet this approach requires knowledge about the genes in these pathways, which is not available for all species, especially not for newly discovered bifidobacterial (sub)species [27].

Recently, we have shown that protein sequence-based subclustering of GH families can delineate enzymes with different substrate specificities [28]. These subtle functional differences are particularly important for deciphering HMG-metabolic potentials between species and conspecific strains, as shown for *B. breve* and *B. longum* [5,29, 30].

To overcome these limitations, we developed *bifidoAnnotator*, a specialized annotation tool providing fine-grained functional annotation of bifidobacterial GH gene repertoires associated with HMG degradation. The tool performs three key functions: (1) annotates GH-encoding genes at the family level, (2) assigns query sequences to fine-grained clusters of homologous proteins with known or predicted functions and (3) generates publication-ready visualisations for comparative analysis. We show the tool’s superior performance using an established gold standard dataset and demonstrate its utility using bifidobacterial genomes from the Amsterdam Infant Microbiome Study (AIMS).

## Theory and implementation

### Reference database

We constructed a comprehensive reference database [bifidoAnnotator database (*bifDB*); Table S1, available in the online Supplementary Material] of bifidobacterial GH-encoding genes associated with HMG utilization from 2,333 genomes and metagenome-assembled genomes (MAGs) spanning 87 bifidobacterial species downloaded from ProGenomes3 [31]. GH-encoding genes were annotated using run_dbCAN4 and run_dbCAN2 implemented in anvi'o (v8 [32]) and filtered to only retain proteins from GH families associated with HMO and *N*-glycan utilization: GH2, GH5_18, GH18, GH20, GH29, GH33, GH38, GH42, GH85, GH95, GH112, GH125 and GH136 [5,33], yielding 24,334 protein sequences. GH families primarily associated with non-HMG substrates were excluded, including GH13, which, despite being abundant in bifidobacterial genomes, is predominantly involved in starch and *α*-glucan metabolism rather than HMG degradation [34].

Protein sequences were clustered per GH family using MMseqs2 (Many-against-Many sequence searching; Fig. S1)) easy-cluster (--min-seq-id 0.70–0.75, -c 0.80, –cov-mode 2, –cluster-mode 0) [35]. Phylogenetic trees were constructed for each GH family using mafft [36] and fasttree [37] to validate clustering and identify misannotations. Trees were inspected to verify that MMseqs2 clusters corresponded to monophyletic or near-monophyletic clades. Cases where a sequence was assigned to one cluster by MMseqs2 but grouped phylogenetically with a different cluster in the GH family tree were flagged for re-evaluation. Minimum cluster size thresholds were determined by examining the cluster size distribution after MMseqs2 clustering of the initial 24,334 GH sequences (Fig. S1A). After evaluating the effect of thresholds ranging from 2 to 10 on the number of clusters and sequences removed (Fig. S1B), a threshold of 5 was chosen. Clusters with fewer than five sequences represented ~61% of all clusters but only ~2% of total sequences (Fig. S1C), indicating that they are numerous but collectively contribute negligible sequence diversity. These underrepresented clusters are more likely to reflect sequencing errors, misannotations or rare variants rather than functionally coherent groups. Clusters meeting this minimum were subsequently validated through phylogenetic analysis and cross-referencing with eggNOG (evolutionary genealogy of genes: Non-supervised Orthologous Groups) [38] and Paperblast [39], a tool that links protein sequences to their mentions in the published literature. In short, sequences or clusters were removed when: (1) both databases showed low identity (<60%) to target GH families, (2) eggNOG annotations indicated non-CAZy functions inconsistent with Paperblast results, or (3) phylogenetic analysis revealed aberrant branching patterns suggesting misannotation.

Each cluster was manually curated using literature evidence to assign a validation status (experimentally validated, putative function or hypothetical). Clusters were classified as ‘experimentally validated’ when at least one member enzyme had published biochemical characterization demonstrating enzymatic activity or substrate specificity; ‘putative function’ when homology-based annotation suggested a likely function but no direct experimental evidence was available; or ‘hypothetical’ when no functional inference could be made beyond GH family membership. Each cluster was additionally assigned an HMG-association status (yes/no/unknown). HMG-association status distinguishes clusters containing enzymes with demonstrated activity on HMG substrates (‘yes’) from non-HMG-utilizing clusters (‘no’), defined as enzymes that, despite belonging to GH families containing HMG-active members, function on alternative substrates other than HMGs. For example, within GH42, some clusters contain *β*-galactosidases active on HMG substrates, while others act on galactooligosaccharides [23]. These non-HMG clusters are retained in *bifDB* to improve annotation accuracy through competitive mapping. This reduces false-positives (FPs) from queries that would otherwise map to HMG-associated clusters with low confidence. Clusters lacking experimental evidence for substrate specificity were assigned ‘unknown’. Subcellular GH localization (intracellular/extracellular) was assigned based on literature evidence and validated using SignalP 6.0 (--mode fasta [40]) on GH protein sequences. Sequences with discordant predictions were evaluated using OD-seq (Outlier Detection in multiple SEQuence alignment) [41] to identify MSA outliers (Table S2), which were subsequently removed from *bifDB*; remaining discordant sequences were found to have N-terminal truncations upon manual inspection. Ultimately, the *bifDB* contained 22,580 bifidobacterial GH sequences (Tables S1 and S3).

### Conserved genomic neighbourhood analysis

A similar genomic neighbourhood can be indicative of functional overlap, even when key enzymes have a moderate degree of divergence. Hence, we performed gene neighbourhood analysis using a modified GeneGrouper workflow [42] to further refine bifidoAnnotator GH cluster boundaries. We selected 176 reference genomes covering all *bifidoAnnotator* GH clusters with five or more sequences. For each of the 13 GH families, we constructed a separate blast database using the GeneGrouper *build* function. Since GeneGrouper accepts only a single reference sequence per run, we ran it iteratively for each GH family, using one representative sequence per GH cluster as the reference (i.e. *seed*) in each run. Homology thresholds for *seed* gene detection were set to match the cluster-specific identity thresholds used in *bifidoAnnotator* (Table S1). This ensured that all GH clusters within a family were queried against the selected reference genomes. Flanking regions of 20 kb upstream and downstream of each GH gene were analysed. Since the same genomic locus could be identified by queries from multiple clusters (e.g. if two genes with the same GH annotation were located next to each other), redundant detections were removed before grouping, ensuring each locus was represented only once.

The deduplicated loci were then grouped by GeneGrouper based on shared flanking gene content. When GH sequences from different clusters within the same family and encoding enzymes with similar predicted functions were grouped together based on conserved gene neighbourhoods, the corresponding *bifidoAnnotator* clusters were merged, as a shared genomic context provides strong evidence for functional orthology (Figs S2–S14). Additionally, GH clusters assigned to different GeneGrouper groups were merged when their filtered gene complements differed by ≤2 genes, as assessed from the gene presence/absence heatmaps. This genomic context-based merging reduced the total number of GH clusters from 122 to 108, consolidating functionally equivalent variants that differed only in sequence identity but not in genomic organization (Table S4). Clusters retained as separate entities within the same GH family may therefore represent functionally equivalent orthologues whose sequence divergence reflects differences in enzymatic properties, such as substrate specificity or kinetic parameters, rather than unrelated functions.

### Threshold optimization

To account for the speed of evolution of different GH families, we optimized sequence per cent identity thresholds for GH family and cluster annotations using MMseqs2. For GH family annotation, we identified for each sequence: (1) the best hit within its assigned family and (2) the best hit outside its family. Optimal thresholds for per cent identity were determined as the values yielding the maximal F1 scores, where F1 = 2 × (precision × recall) / (precision + recall), with sequences assigned to the other 12 GH families serving as negative examples (Figs S15–S17).

For protein cluster assignment within GH families, we implemented a percentile-based approach for per cent identity thresholds. For each protein cluster, we calculated the distribution of per cent identity values from intra-cluster matches and set thresholds at the tenth percentile (Fig. S18). The tenth percentile was chosen as a conservative convention to ensure that the large majority of within-cluster matches would exceed the threshold while allowing for natural sequence variation within clusters. To ensure hierarchical consistency, cluster assignment thresholds were set as the maximum of either the family threshold or the percentile-derived value, ensuring that cluster assignment criteria were always at least as stringent as GH family assignment criteria (Table S1). Cutoffs for coverage (50%) and bitscore (200) were found to yield high accuracy in conjunction with the optimized per cent identity thresholds for both GH family and cluster assignments.

### Transporter annotation module

To support interpretation of GH annotations in the context of HMG utilization pathways, we implemented a transporter annotation module. We curated a reference database of 103 protein sequences representing 52 distinct HMG-associated transporter genes from the literature [5,20, 26], covering transport systems for HMOs, *N*-glycans and products of extracellular GH activity (bifidoAnnotator transporter protein datbase,*bifTPDB*; Table S5). Transporter annotation is performed using MMseqs2 with a sequence identity threshold of 80%. The module enables users to assess whether genomes carrying intracellular- and/or extracellular-acting GHs also harbour associated transport systems, providing an additional line of evidence for inferring HMG utilization potential beyond GH repertoire alone. To illustrate the module’s utility, transporter annotations were inspected across all 147 *B. breve* genomes present in *bifDB*.

### Implementation and annotation workflow

**Fig. 1.**
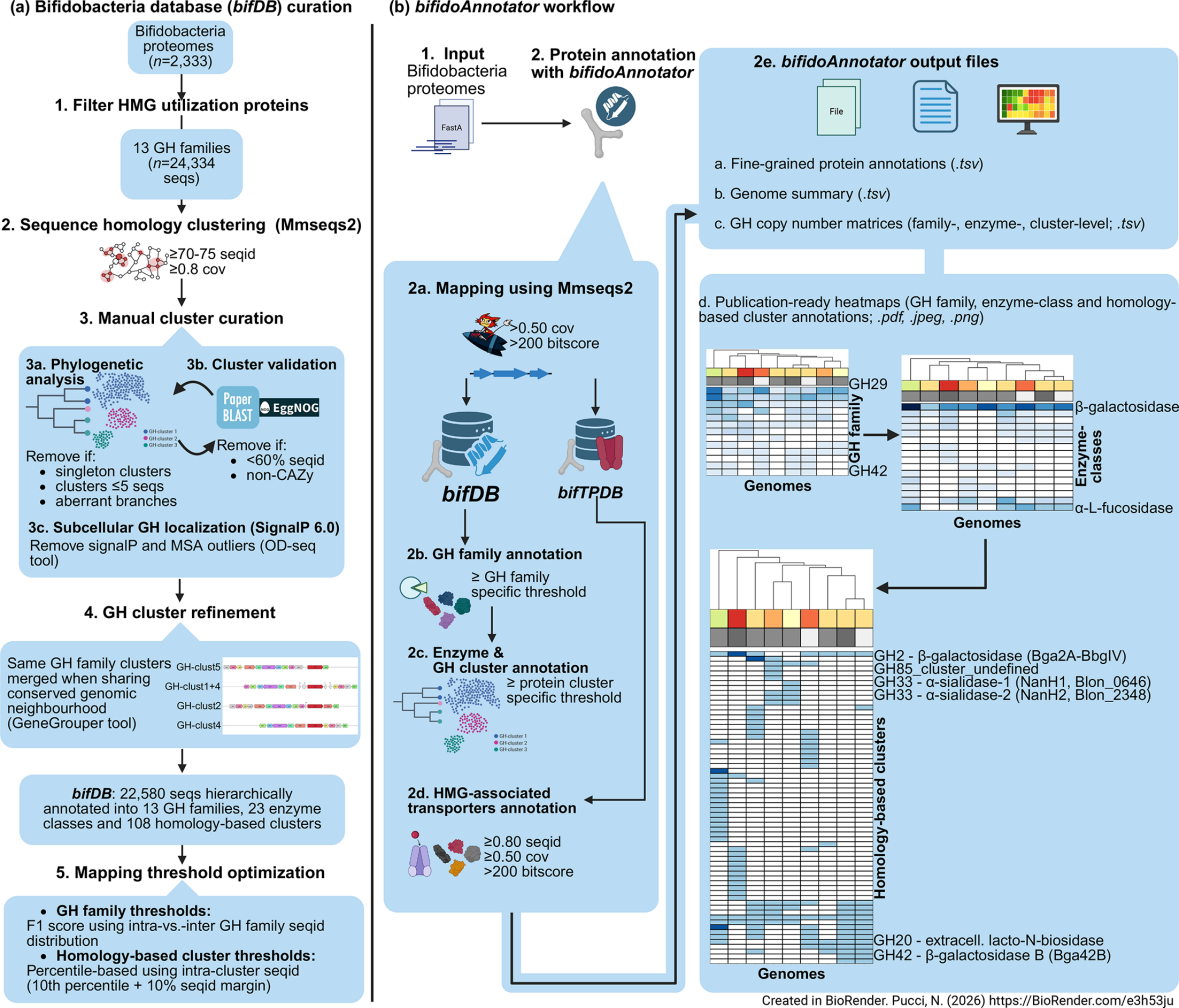
*bifDB* curation and workflow overview. (**a**) *bifDB* curation: HMG utilization proteins from 2,333 bifidobacterial proteomes were clustered using MMseqs2 and manually curated, yielding 22,580 sequences across 108 homology-based clusters. (**b**) *bifidoAnnotator* workflow: bifidobacterial sequences are mapped against *bifDB* using MMseqs2 for GH family and cluster-level annotation. Additionally, bifidobacterial sequences are mapped against *bifTPDB,* a database of bifidobacterial HMGs transporters. Output includes detailed annotation tables, copy number matrices and publication-ready heatmaps. cov, coverage; seqs, sequences.

*bifidoAnnotator* is implemented as a Python program and is distributed as a conda package. It accepts protein sequences in FASTA (Fast-All) format and a sample file with genome identifiers for output naming. *bifidoAnnotator* supports single-file and batch processing modes and performs hierarchical annotation through a two-stage process. First, it maps query protein sequences to the reference databases (*bifDB* and, optionally, *bifTPDB*) via MMseqs2 (Fig. 1). Query sequences are classified into specific GH families when their best database hit meets the GH-specific threshold. Subsequently, *bifidoAnnotator* attempts cluster assignment within that family, if the query’s best hit meets cluster-specific thresholds for per cent identity. Query proteins receiving GH family annotation but failing cluster criteria are assigned ‘cluster undefined’.

### Output files

*bifidoAnnotator* generates a structured output directory with multiple output formats (Fig 1):

- *bifidoAnnotator_tables*: detailed annotation tables with GH family, enzyme and GH cluster assignments, predicted subcellular localization, validation status, HMG-association status, transporter annotations and mapping statistics.
- *bifidoAnnotator_visualizations*: three publication-ready heatmaps, showing protein copy number at family, enzyme class and GH cluster levels.
- The --annotation_file parameter accepts tab-separated (TSV) files with genome metadata displayed as heatmap column annotations. A row annotation bar indicating HMG-association status is included in the GH cluster heatmap, which can be filtered using --hmg-unknown (default; ‘yes’ and ‘unknown’ clusters), --hmg-only (‘yes’ only) or --all-genes (all clusters).

### Performance evaluation

We benchmarked *bifidoAnnotator* against six established annotation tools [run_dbCAN2 (database for automated Carbohydrate-active enzyme ANnotation 2) [43], run_dbCAN3, run_dbCAN4 [44], eCAMI, CUPP (Conserved Unique Peptide Patterns) [45] and bifidotyper (https://github.com/Bennibraun/bifidotyper)] using nine bifidobacterial genomes with expert-curated HMG utilization gene repertoires [5]: *B. longum* subsp. *infantis* (strain ATCC 15697), *B. longum* subsp. *longum* (JCM 1217 and SC596), *B. longum* subsp. *suis* (JDM 301), *B. breve* (UCC 2003), *B. bifidum* (PRL 2010), *Bifidobacterium catenulatum* subsp. *kashiwanohense* (JCM 15439), *Bifidobacterium pseudocatenulatum* (JCM 1200) and *Bifidobacterium scardovii* (JCM 12489). To assess broad GH family annotation performance, we performed identical benchmarking using CAZy db (carbohydrate-acting enzyme database) as the reference dataset. Runtime was measured by summing processing time across the dataset. Bifidotyper was evaluated on five genomes due to the limited availability of compatible input data.

Additionally, GH95 cluster assignments were validated against 12 strains with experimentally characterized extracellular (*B. bifidum* ATCC 29251, *n*=4) or intracellular (*B. breve* UCC2003, *Bifidobacterium infantis* ATCC 15697, *B. longum* SC596, *B. pseudocatenulatum* SC586 and MP80, *Bifidobacterium kashiwanohense* JCM15439, PV20-2 and APCKJ1) *α*-l-fucosidases [46,50].

We demonstrated *bifidoAnnotator*’s capabilities using 49 high-quality (≥90% completeness, ≤5% contamination) bifidobacterial MAGs from stool and oral samples of mothers (34 weeks gestation) and infants (1- and 6-month-old) from the AIMS (Table S6) [28]. Species, sampling timepoint and feeding metadata were given as input for graphical outputs.

## Results

### Bifidobacterial protein database

Our curated database contains 22,580 sequences from 2,309 genomes (mean copy number sd=9.8±3.9; range=1–25), representing 13 GH families across 79 bifidobacterial species. MMseqs2 clustering yielded 122 GH clusters, with cluster sizes ranging from 4 to 2,062 sequences (median=73; Table S1). Genomic context analysis further consolidated the 122 clusters into 108 (Table S3), by merging clusters that shared conserved gene neighbourhoods (Figs S2–S14, Table S4).

The largest GH families were GH2 (*n*=5,949, clusters=37), GH42 (*n*=5,526, clusters=15), GH38 (*n*=2,325, clusters=8) and GH20 (*n*=2,038, clusters=12), accounting for 70% of total sequences. Manual curation assigned validation status to all clusters: 35.1% (38 out of 108) contained experimentally validated enzymes, 49.0% (53 out of 108) had homology-based functional annotations, and 15.7% (17 out of 108) remained hypothetical (Table S1).

### Performance evaluation

**Fig. 2.**
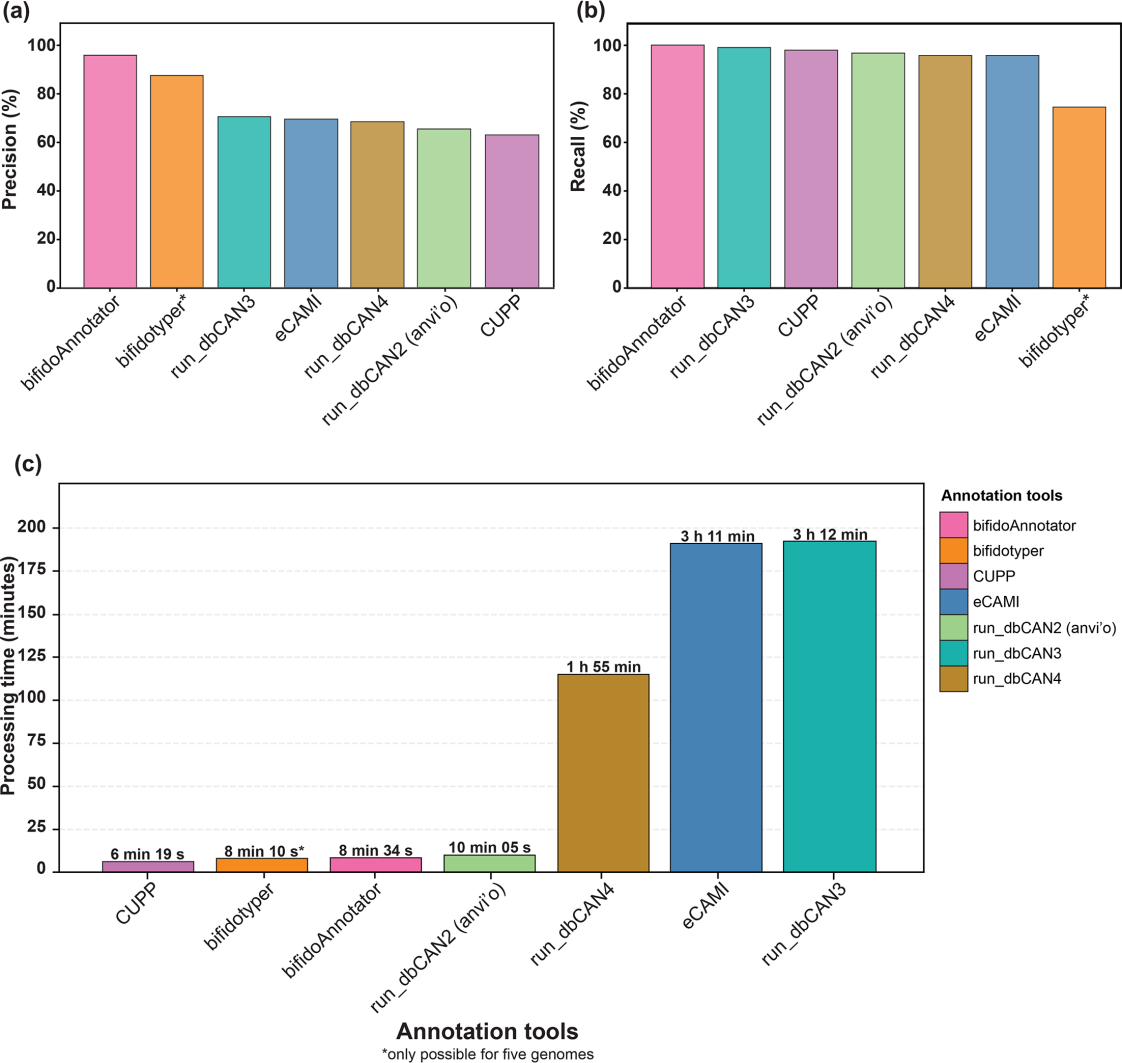
Performance comparison of bifidoAnnotator vs. state-of-the-art annotation tools. Bar plots showing (**a**) precision and (**b**) recall metrics for functional cluster annotation and (**c**) processing time. Tools (bifidoAnnotator=pink, bifidotyper=orange, CUPP=purple, eCAMI=blue, run_dbCAN2=green, run_dbCAN3=water green and run_dbCAN4=beige) were evaluated on nine bifidobacterial genomes with manually curated HMG utilization gene repertoires, using the Arzamasov and Osterman [5] dataset as the gold standard reference for comparison. Bars are ordered by performance values. Precision and recall represent the accuracy of each tool’s functional cluster annotations when compared against the gold standard. N.B. bifidotyper performance is based on only five genomes. N.B., nota bene

**Fig. 3.**
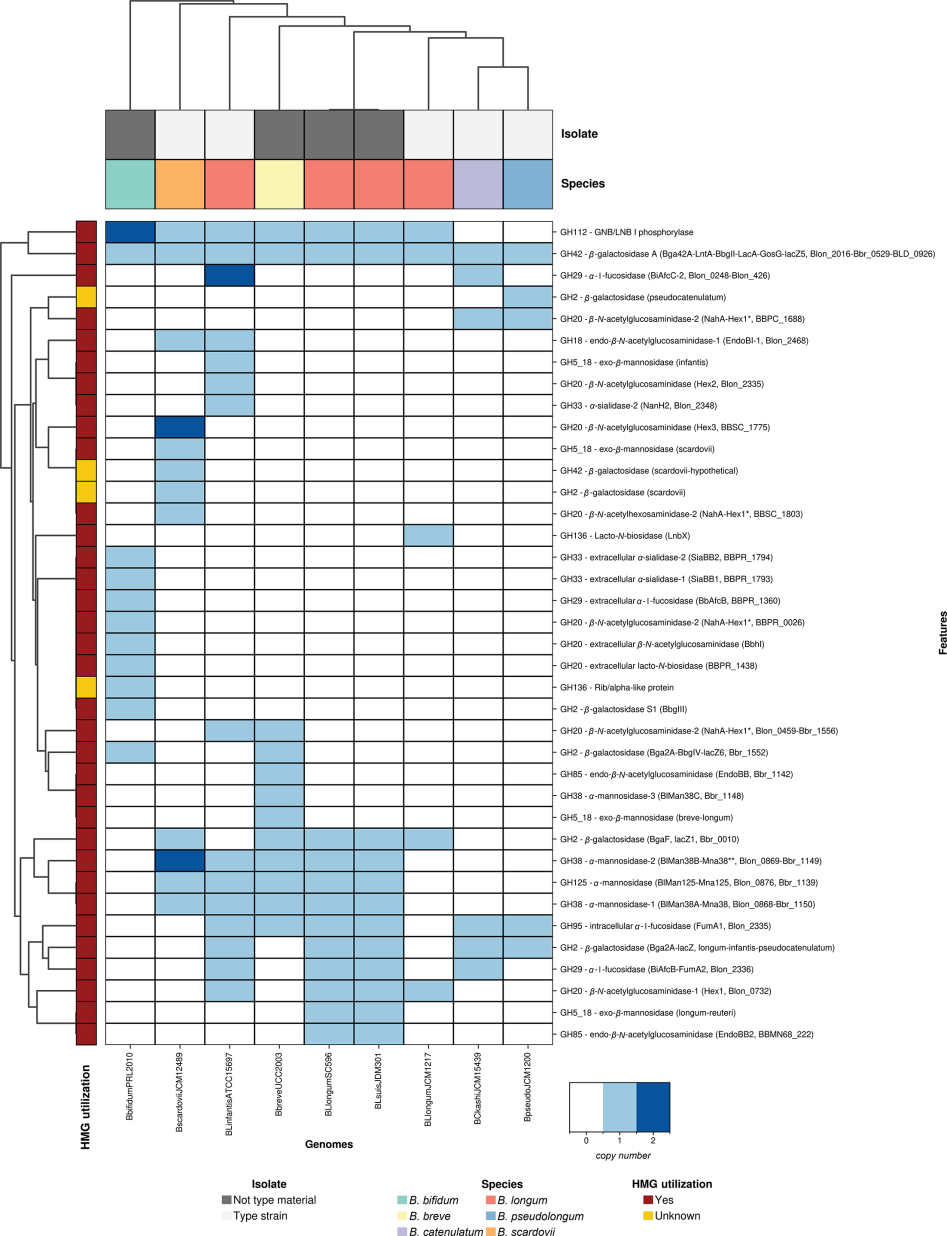
*bifidoAnnotator* reveals species- and strain-level diversity in HMG utilization gene repertoires across bifidobacterial genomes. Heatmap showing copy numbers (white-to-blue scale) of functional clusters (rows) across nine well-characterized publicly available bifidobacterial genomes (columns). Genomes are hierarchically clustered with annotation bars indicating species and isolate-type material designation (isolate: grey scale). Cluster-level resolution reveals inter- and intra-specific variation in HMG utilization genes, including distinct enzyme variants within the same GH family masked by traditional family-level annotations. Species represented: *B. bifidum* (green), *B. breve* (yellow), *B. catenulatum* (purple), *B. longum* (red, including the subspecies *BL infantis*, *BL longum* and *BL suis*), *B. pseudocatenulatum* (blue) and *B. scardovii* (orange). BL, *B. longum*; GNB, galacto-N-biose; LNB, lacto-N-biose.

We compared *bifidoAnnotator* and six established carbohydrate-active enzyme (CAZyme) annotation tools (see Methods) (Table S7) [5]. *bifidoAnnotator* achieved the highest precision among tools [95.9%, true-positive (TP)=93, FP=4; Fig. 2a] while maintaining 100% recall (TP=93, false-negative=0; Fig. 2b, Table S8). The higher precision of *bifidoAnnotator* compared with dbCAN tools reflects its cluster-level resolution, which distinguishes functionally distinct variants within the same GH family (e.g. HMO-utilizing vs. non-HMO-utilizing enzymes within GH2 and GH33), a distinction that family-level tools do not capture (Tables S9 and S10, Fig. 3).

Manual inspection of *bifidoAnnotator* results revealed three GH2 and one GH112 annotations as FPs (i.e. genes not associated with HMG metabolism by Arzamasov and Osterman [5]), yet all of them were (near) identical (99.5–100% sequence identity, seqid) to experimentally validated proteins for lactose, LNnT (GH2 *β*-galactosidase variant BgaF [23]) and GNB/LNB I (galacto-N-biose/lacto-N-biose) phosphorolysis (GH112 – GNB/LNB I phosphorylase [51]; Table S11). Performance analysis using CAZy db as the gold standard for broad-level GH family annotation also showed *bifidoAnnotator* to be the best-performing tool, followed closely by run_dbCAN2 and run_dbCAN3 (Fig. S19, Table S12).

To further validate cluster assignments, we cross-referenced *bifidoAnnotator* annotations against additional strains with experimentally characterized enzymes from the literature. As an illustrative example, GH95 cluster assignments were verified against 12 strains carrying either extracellular or intracellular *α*-l-fucosidase (see Methods), as these two functionally distinct variants are well-characterized in the literature [46,50]. As expected, all *B. bifidum* genomes carried the extracellular cluster (GH95 – extracellular *α*-l-fucosidase, protein AfcA), while all other species mapped exclusively to the intracellular cluster (GH95 – intracellular *α*-l-fucosidase, FumA1) (Table S13).

To demonstrate the utility of the transporter annotation module, we inspected transporter annotations across all 147 *B. breve* genomes in *bifDB* (Tables S14 and S15). The majority of genomes carried transporters associated with HMO (HMO transporterGltA/B/C: 99–100%), beta-galactoside (GosABC: 95–99%), sialic acid (NanBCDF: 98%) and *N*-glycan substrates (MnaA: 64%), while GlcNAc transporters (protein NagE2a/bc) were detected in 70.7% of genomes (Fig. S20). Notably, while all 147 genomes carried the fucose permease (FucP) required for free fucose import and the GH95 intracellular *α*-l-fucosidase gene (Table S14), only 27.2% harboured the associated fucosyllactose 2 (FL2) transporter for fucosylated HMOs, illustrating how transporter annotation reduces overestimation of HMG utilization capacity based on GH repertoire alone.

Benchmarking was performed on a server with 1 core and 90 GB of random access memory. Runtime analysis demonstrated that *bifidoAnnotator* is orders of magnitude faster than several established tools. *bifidoAnnotator* (8 min 34 s) completed processing in comparable time to outperform most tools except CUPP (6 min 20 s), while run_dbCAN3 (192 min), eCAMI (191 min) and run_dbCAN4 (115 min) required substantially longer processing times (Fig. 2c), representing speed improvements of 22-fold or greater. Bifidotyper processed five genomes in 8 min 11 s.

### *bifidoAnnotator* reveals strain-level variation and functional specialization in HMG utilization

To demonstrate *bifidoAnnotator’s* utility, we analysed 49 bifidobacterial MAGs from eight species retrieved from stool and oral samples of mothers (34 weeks gestation) and infants (1- and 6-month-old) from the AIMS (Table S6) [28].

All bifidobacterial MAGs carried HMG-metabolizing genes (Fig. S21). *B. infantis* had the highest copy number of HMG-associated GH genes (19.1±1.9), followed by *B. bifidum* (18.0±2.0) and *B. breve* (15.5±2.1). This ranking is consistent with current literature recognizing these three species as the primary HMG utilizers among bifidobacteria [5,7]. To evaluate *bifidoAnnotator’s* ability to detect known functional differences between bifidobacterial species, we examined cluster-level annotations revealing enzyme variants masked by broad family classifications. For instance, cluster analysis of GH33 sialidases revealed *B. bifidum* to carry the extracellular-acting sialidases SiaBB1/SiaBB2, while *B. infantis* possessed an intracellular-acting *α*-2,3/6-sialidase A protein (NanH1). By combining the genomic results with host information for the recovered MAGs, we found the GH29 BiAfcC-2 *α*-l-fucosidase cluster, associated with intracellular fucosylated HMO utilization, to be exclusively detected in *B. infantis* MAGs from 6-month-old infants (4/10 MAGs; Fig. S23), suggesting possible age-related strain-level specialization in fucosylated HMO metabolism. The tool also distinguished known GH2 and GH42 non-HMG-utilizing clusters [23,52] and revealed intra-specific variation in *α*-mannosidases (GH38, GH125) and endo-*β*-*N*-acetylglucosaminidases (GH85) across *Bifidobacterium adolescentis* and *B. longum* strains. Overall, *bifidoAnnotator’s* cluster-level resolution reveals functional diversity consistent with previous findings, demonstrating utility for comparative genomics and metabolic profiling (Figs S21–S26, Tables S16–S20).

## Conclusion

*bifidoAnnotator* provides accurate, user-friendly and efficient annotations of bifidobacterial HMG utilization potential using a large, curated database of GH sequences.

Benchmarking demonstrated competitive performance, achieving superior precision compared with established state-of-the-art tools while maintaining fast annotation speed, being an order of magnitude faster than the most accurate established annotation tools. Traditional GH family-level annotations mask the underlying functional diversity [24,25], which *bifidoAnnotator* reveals by further subclustering the families. Combined with efficient processing and publication-ready visualizations, this enables researchers to detect and contextualize bifidobacterial species- and strain-level variation in carbohydrate metabolism at scale. For instance, applied to the AIMS cohort, *bifidoAnnotator* recapitulated known species-level specializations, such as the differential distribution of sialidase variants across *B. bifidum* and *B. infantis* [18,53,55]. The tool also revealed intra-specific variation in *N*-glycan-associated enzymes and fucosylated HMO metabolism genes across *B. longum* and *B. infantis* strains [5,28], with some variants showing novel associations with infant age. Such patterns, invisible to family-level tools, illustrate how cluster-level annotation enables linking of GH repertoires to biological context (e.g. host age, milk feeding type) and ecological dynamics (e.g. mother–infant strain transmission and early-life gut colonization), providing a foundation for follow-up experimental work.

Even though *bifidoAnnotator* does not perform full pathway reconstruction (as tools such as *glycobif* do [26]), the provided GH and transporter annotations, together with contextual evidence, enable accurate HMG utilization capacity inference. The *B. breve* transporter analysis further illustrates this point; while *FucP* was near-universally detected, consistent with *B. breve*’s known capacity for free fucose utilization [29], the fucosyllactose 1 (FL1) and FL2 transporter prevalence revealed that direct fucosylated HMO utilization is restricted to a functionally distinct subset of strains.

With demonstrated scalability on large metagenomic datasets, *bifidoAnnotator* bridges the gap between broad functional classifications and detailed characterization required for precision microbiome research, representing a valuable addition to the toolkit for studying bifidobacterial genomics.

## Supplementary material

10.1099/mgen.0.001702 Fig. S1.

10.1099/mgen.0.001702 Table S1.

## Abbreviations

AIMS: Amsterdam Infant Microbiome Study
*bifDB*: bifidoAnnotator database
bifTPDB: bifidoAnnotator transporter protein database
CAZyme: carbohydrate-active enzyme
CUPP: Conserved Unique Peptide Patterns
eCAMI: enzyme Classification And Motif Identification
eggNOG: evolutionary genealogy of genes: non-supervised orthologous groups
FL: fucosyllactose
FP: false-positive
GH: glycoside hydrolase
GNB: galacto-N-biose
HMG: human milk glycan
HMO: human milk oligosaccharides
LNB: lacto-N-biose
LNnT: lacto-N-neotetraose
MAG: metagenome-assembled genome
TP: true-positive
