# Supplementary material for: bifidoAnnotator: fine-grained annotation of bifidobacterial glycoside hydrolases for human milk glycan utilization
Source: Microb Genom. 2026 Apr 27;12(4):001702. doi: 10.1099/mgen.0.001702 (PMC13120772; doi:10.1099/mgen.0.001702)
Supplement: Fig. S1. [file mgen-12-01702-s001.pdf]

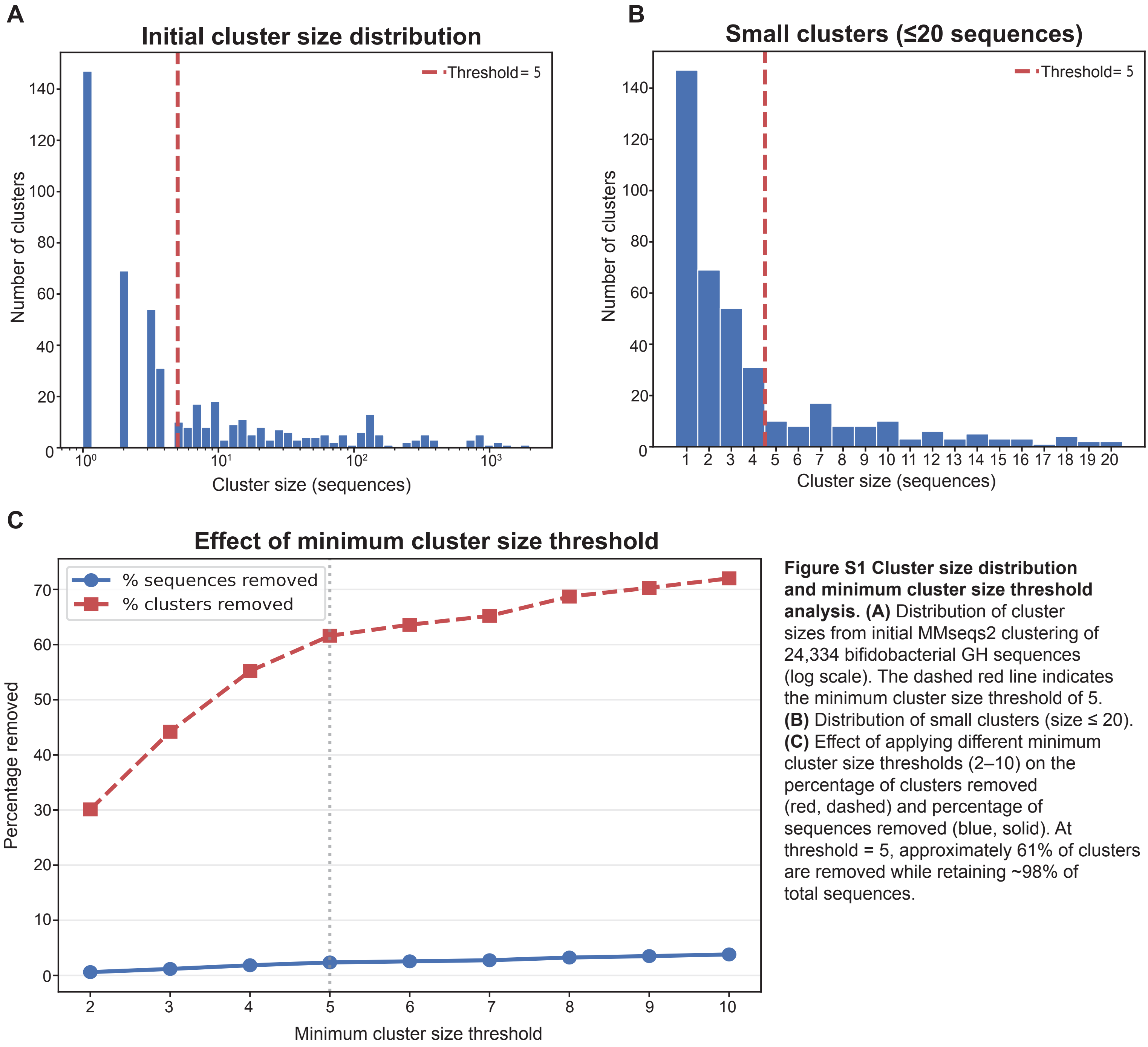

Figure S1 Cluster size distribution and minimum cluster size threshold analysis. (A) Distribution of cluster sizes from initial MMseqs2 clustering of 24,334 bifidobacterial GH sequences (log scale). The dashed red line indicates the minimum cluster size threshold of 5. **(B)** Distribution of small clusters (size ≤ 20). **(C)** Effect of applying different minimum cluster size thresholds (2–10) on the percentage of clusters removed (red, dashed) and percentage of sequences removed (blue, solid). At threshold = 5, approximately 61% of clusters are removed while retaining ~98% of total sequences.

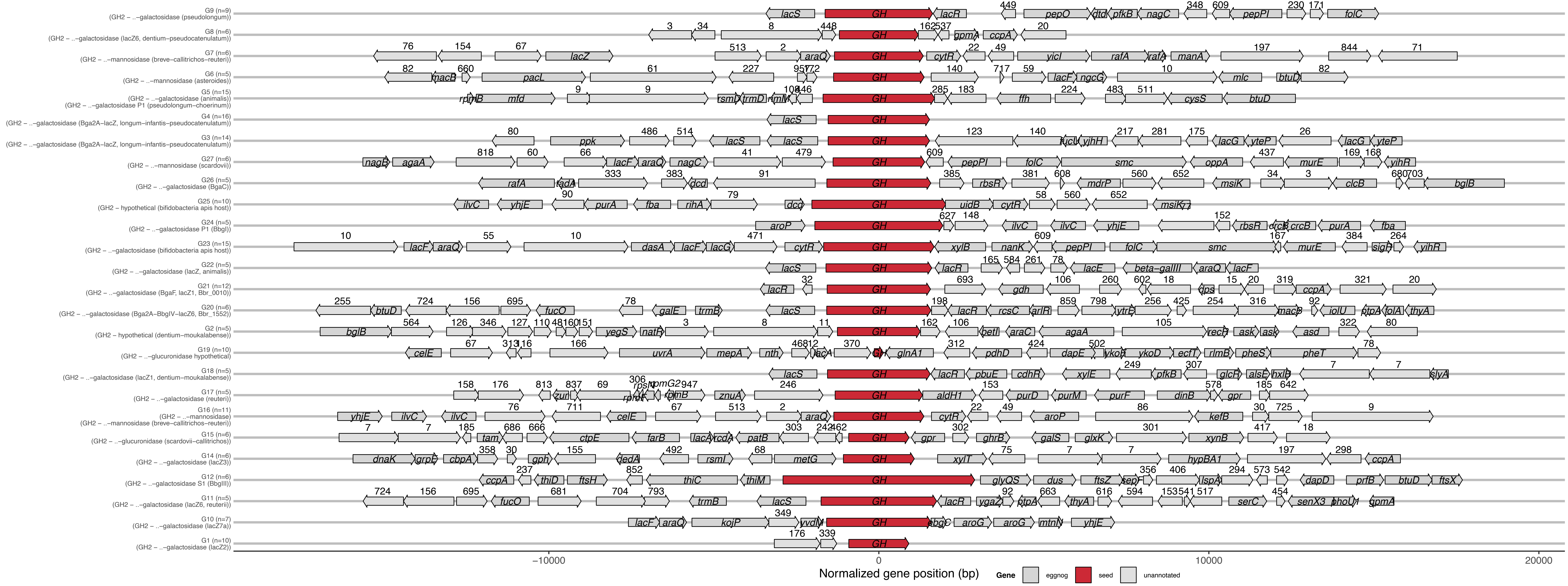

Figure S2. Conserved genomic neighbourhoods of GH2 genes in *Bifidobacterium*. Arrows represent genes colored by annotation source: seed gene (red), reference-matched genes (distinct colors), eggNOG-annotated (dark gray), and unannotated (light gray). Positions are normalized to the seed gene midpoint. Only orthologous clusters present in ≥3 species were retained, with all intervening genes within the outermost shared genes included. Group labels indicate sample size and cluster annotation.

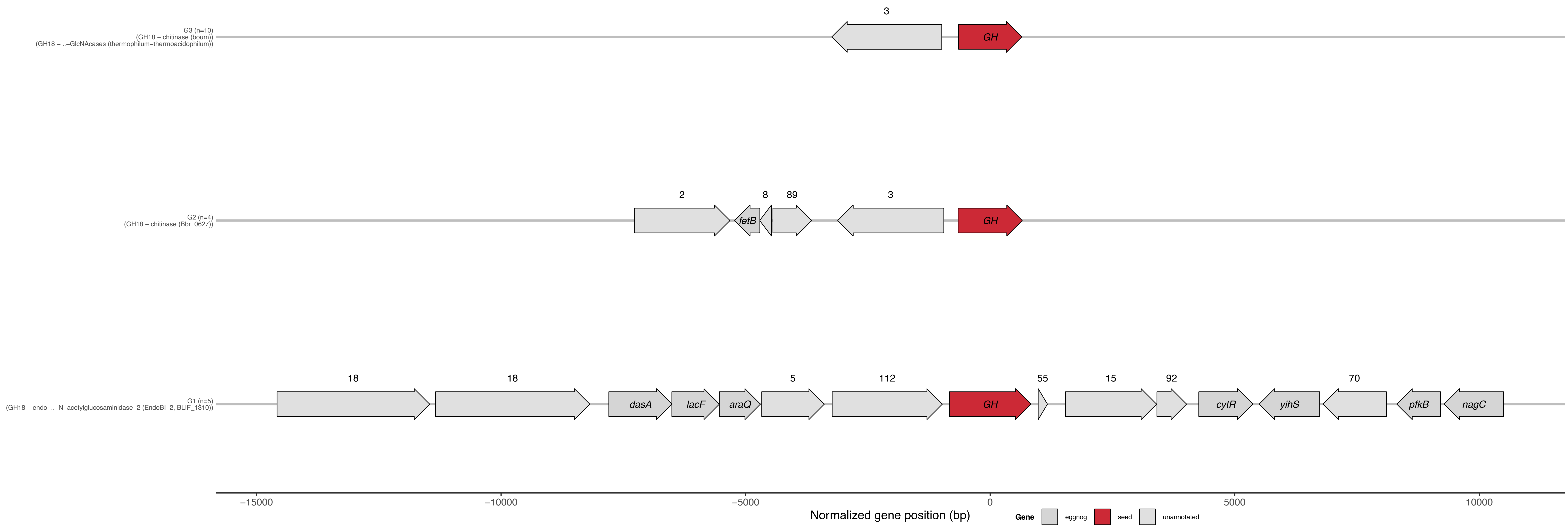

Figure S3. Conserved genomic neighbourhoods of GH18 genes in *Bifidobacterium*. Arrows represent genes colored by annotation source: seed gene (red), reference-matched genes (distinct colors), eggNOG-annotated (dark gray), and unannotated (light gray). Positions are normalized to the seed gene midpoint. Only orthologous clusters present in ≥3 species were retained, with all intervening genes within the outermost shared genes included. Group labels indicate sample size and cluster annotation.

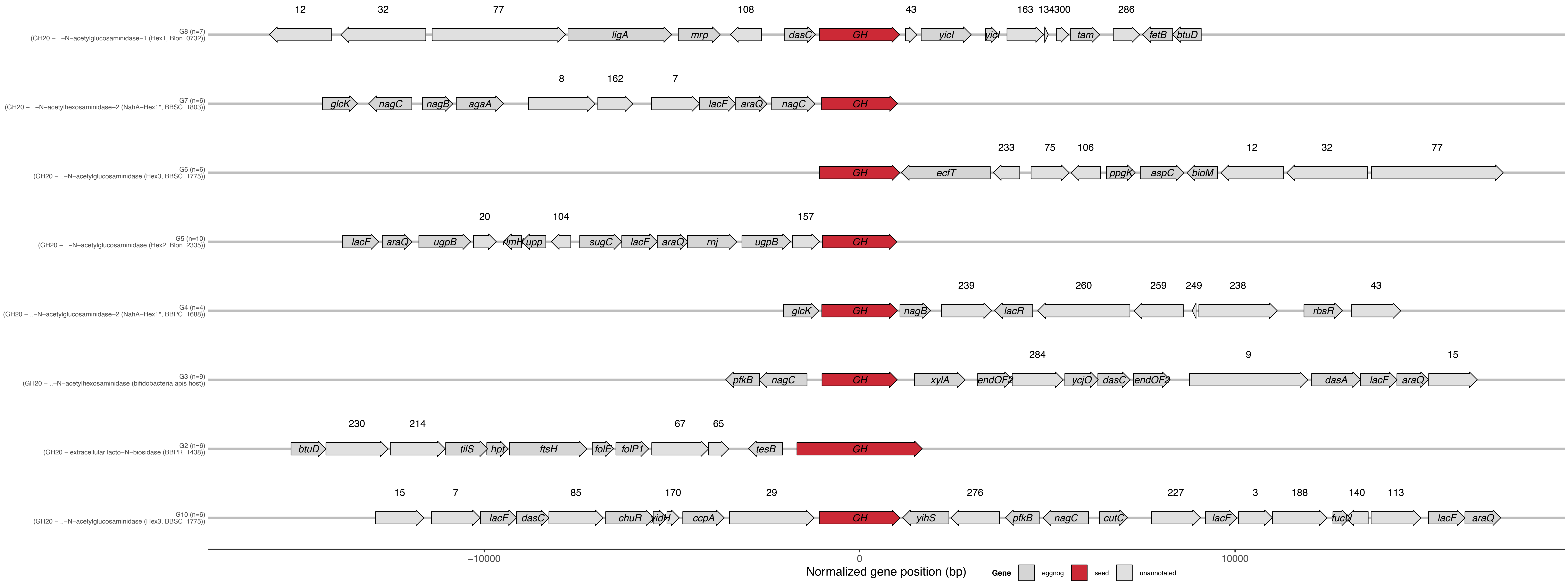

Figure S4. Conserved genomic neighbourhoods of GH20 genes in *Bifidobacterium*. Arrows represent genes colored by annotation source: seed gene (red), reference-matched genes (distinct colors), eggNOG-annotated (dark gray), and unannotated (light gray). Positions are normalized to the seed gene midpoint. Only orthologous clusters present in ≥3 species were retained, with all intervening genes within the outermost shared genes included. Group labels indicate sample size and cluster annotation.

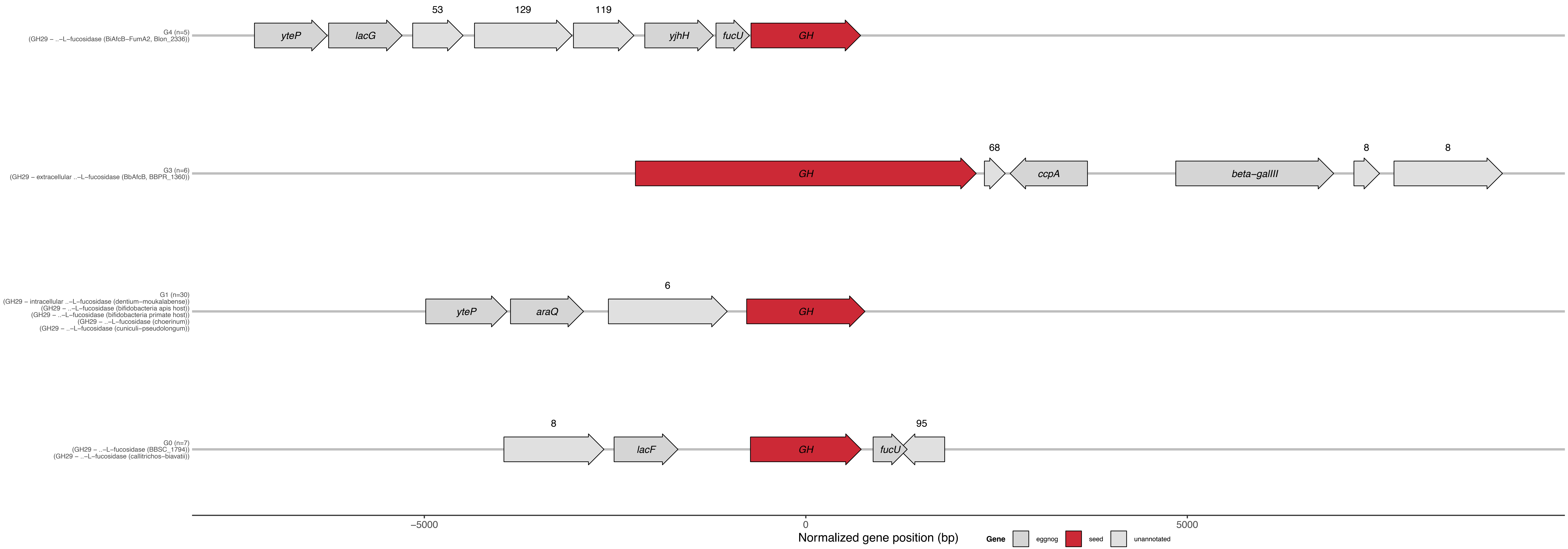

Figure S5. Conserved genomic neighbourhoods of GH29 genes in *Bifidobacterium*. Arrows represent genes colored by annotation source: seed gene (red), reference-matched genes (distinct colors), eggNOG-annotated (dark gray), and unannotated (light gray). Positions are normalized to the seed gene midpoint. Only orthologous clusters present in ≥3 species were retained, with all intervening genes within the outermost shared genes included. Group labels indicate sample size and cluster annotation.

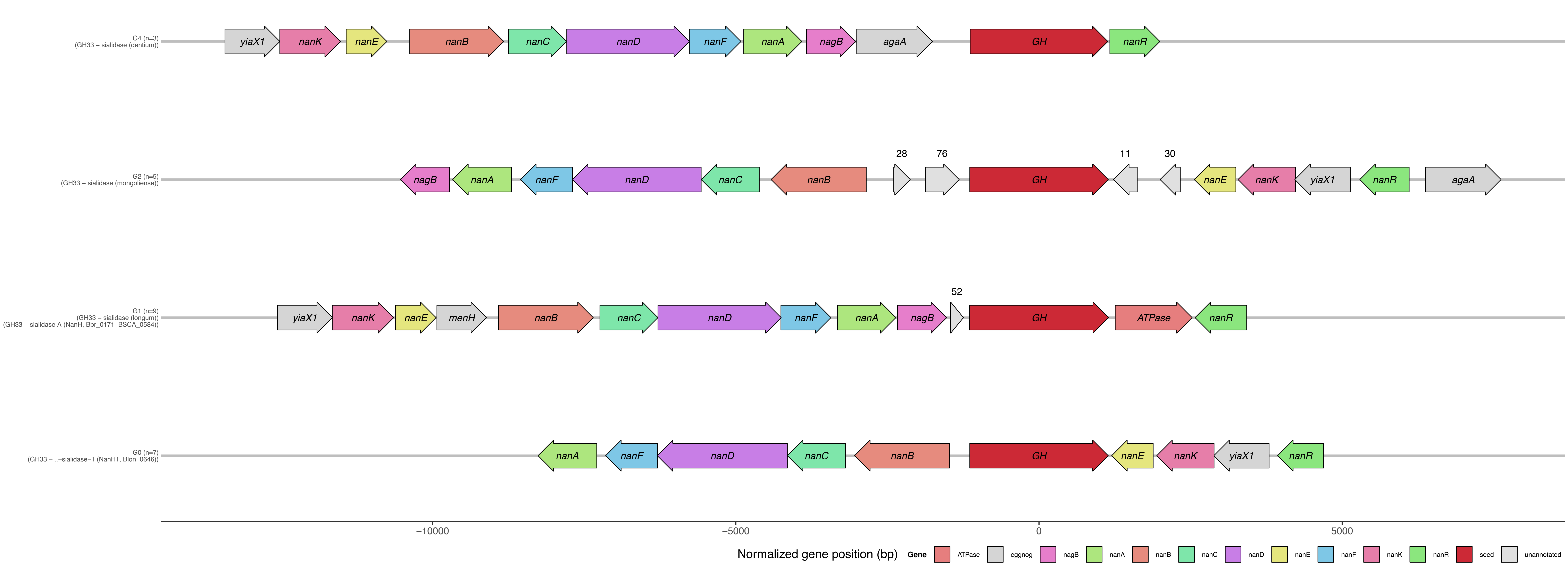

Figure S6. Conserved genomic neighbourhoods of GH33 genes in *Bifidobacterium*. Arrows represent genes colored by annotation source: seed gene (red), reference-matched genes from a curated locus database (distinct colors), reference-matched genes (distinct colors), eggNOG-annotated (dark gray), and unannotated (light gray). Positions are normalized to the seed gene midpoint. Only orthologous clusters present in ≥3 species were retained, with all intervening genes within the outermost shared genes included. Group labels indicate sample size and cluster annotation. For GH33, flanking genes were additionally annotated against characterized *nan* operon components.

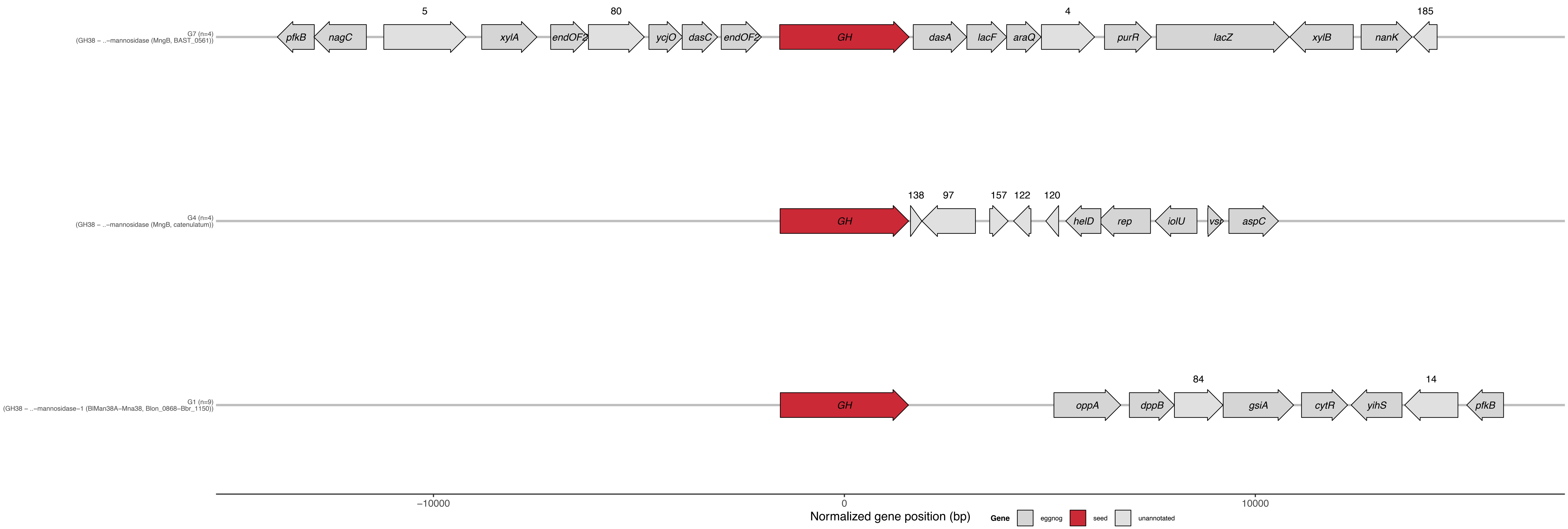

Figure S7. Conserved genomic neighbourhoods of GH38 genes in *Bifidobacterium*. Arrows represent genes colored by annotation source: seed gene (red), reference-matched genes (distinct colors), eggNOG-annotated (dark gray), and unannotated (light gray). Positions are normalized to the seed gene midpoint. Only orthologous clusters present in ≥3 species were retained, with all intervening genes within the outermost shared genes included. Group labels indicate sample size and cluster annotation.

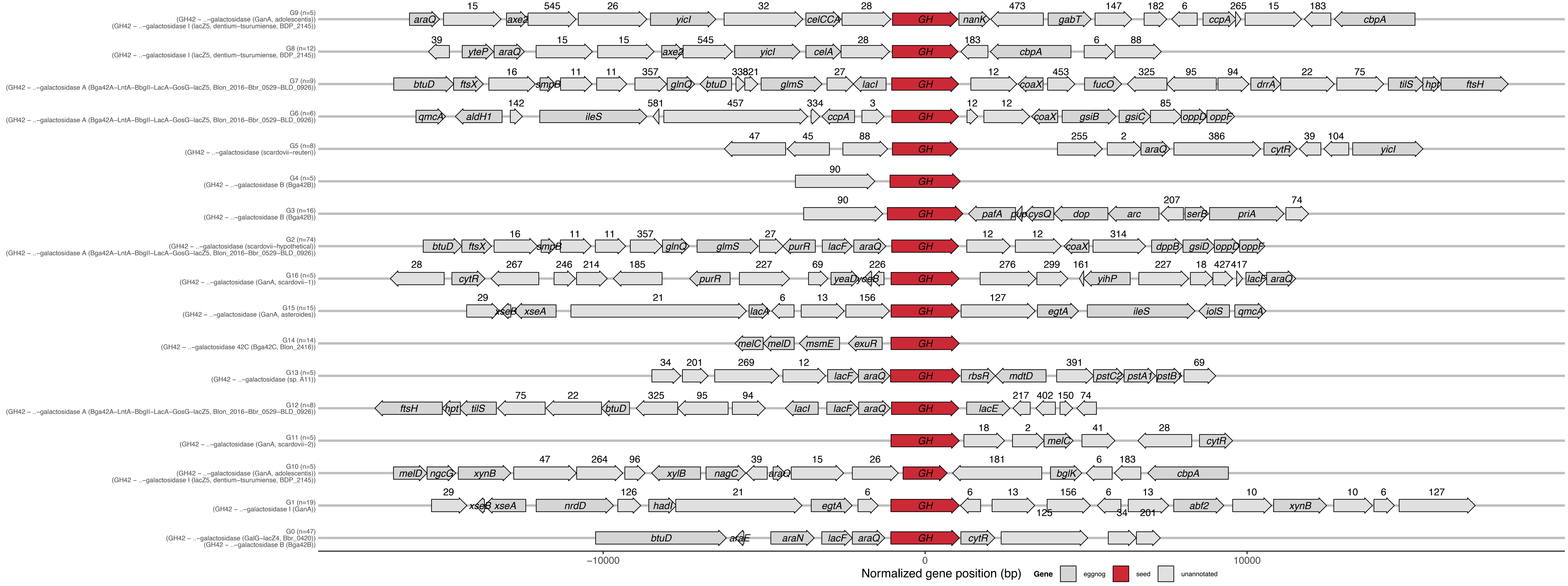

Figure S8. Conserved genomic neighbourhoods of GH42 genes in *Bifidobacterium*. Arrows represent genes colored by annotation source: seed gene (red), reference-matched genes (distinct colors), eggNOG-annotated (dark gray), and unannotated (light gray). Positions are normalized to the seed gene midpoint. Only orthologous clusters present in ≥3 species were retained, with all intervening genes within the outermost shared genes included. Group labels indicate sample size and cluster annotation.

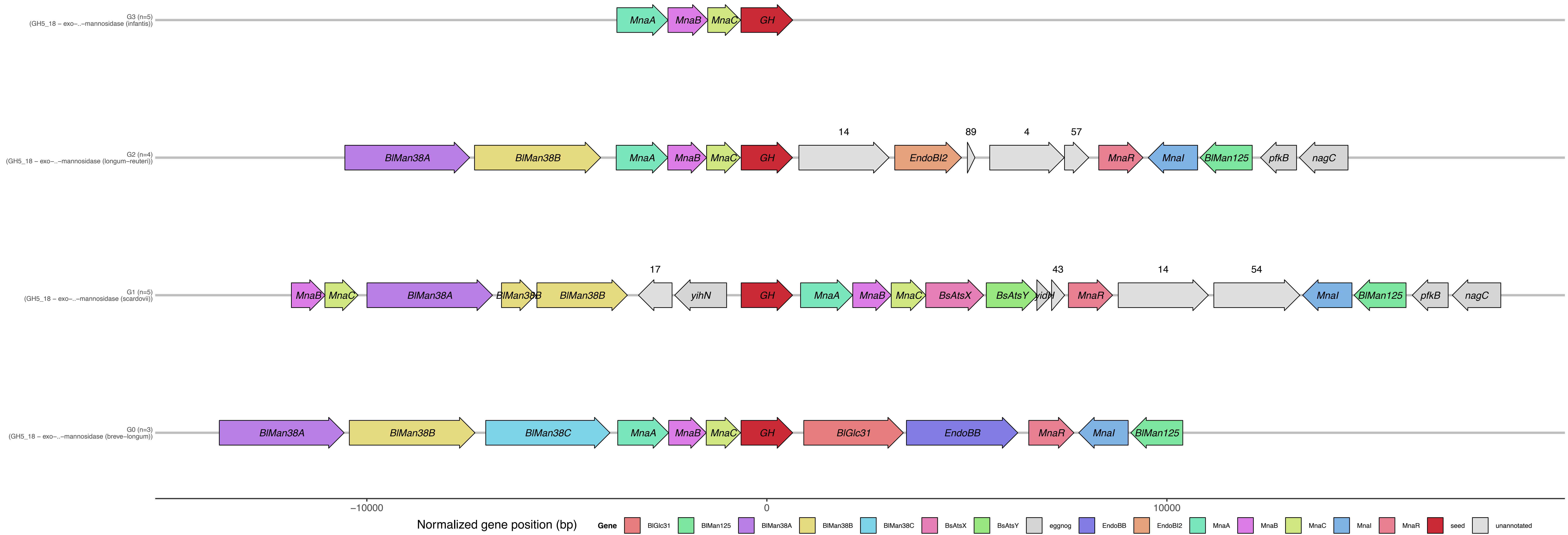

Figure S9. Conserved genomic neighbourhoods of GH5_18 genes in *Bifidobacterium*. Arrows represent genes colored by annotation source: seed gene (red), reference-matched genes from a curated locus database (distinct colors), reference-matched genes (distinct colors), eggNOG-annotated (dark gray), and unannotated (light gray). Positions are normalized to the seed gene midpoint. Only orthologous clusters present in ≥3 species were retained, with all intervening genes within the outermost shared genes included. Group labels indicate sample size and cluster annotation. For GH5_18, flanking genes were additionally annotated against characterized N-glycan utilization locus components.

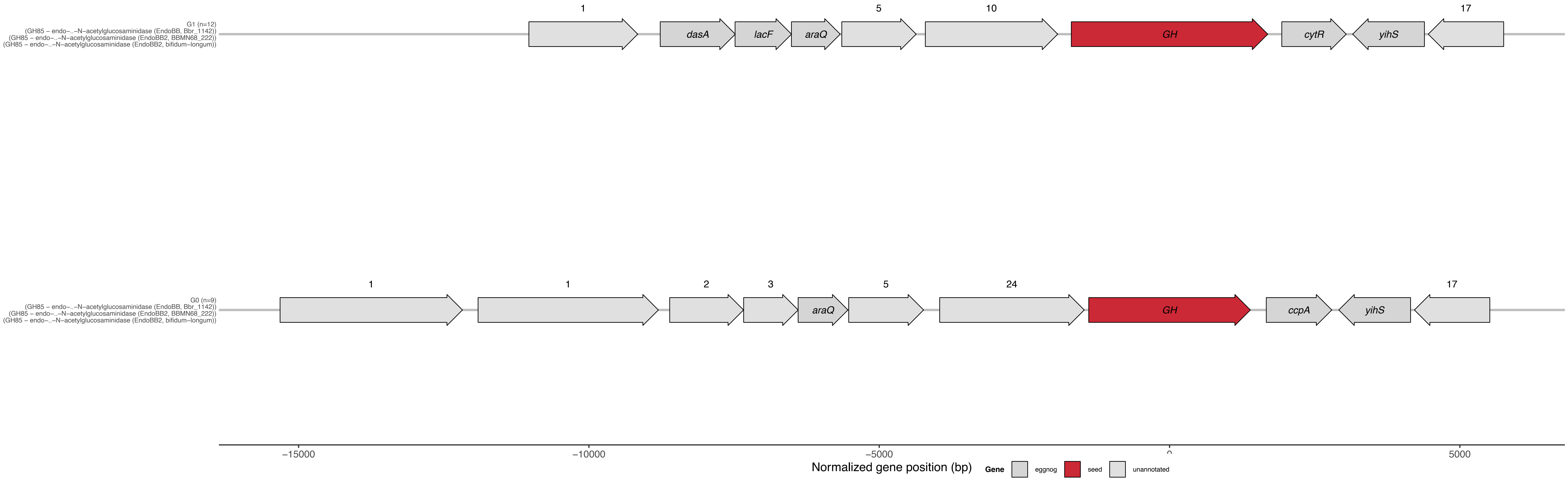

Figure S10. Conserved genomic neighbourhoods of GH85 genes in *Bifidobacterium*. Arrows represent genes colored by annotation source: seed gene (red), reference-matched genes (distinct colors), eggNOG-annotated (dark gray), and unannotated (light gray). Positions are normalized to the seed gene midpoint. Only orthologous clusters present in ≥3 species were retained, with all intervening genes within the outermost shared genes included. Group labels indicate sample size and cluster annotation.

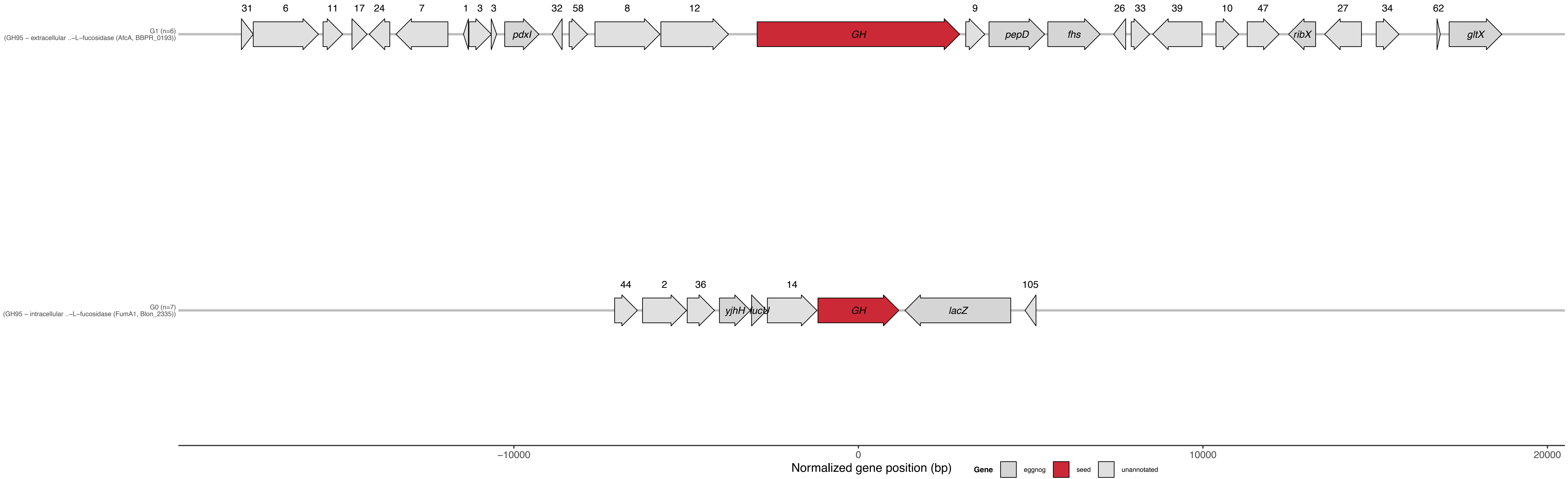

Figure S11. Conserved genomic neighbourhoods of GH95 genes in *Bifidobacterium*. Arrows represent genes colored by annotation source: seed gene (red), reference-matched genes (distinct colors), eggNOG-annotated (dark gray), and unannotated (light gray). Positions are normalized to the seed gene midpoint. Only orthologous clusters present in ≥3 species were retained, with all intervening genes within the outermost shared genes included. Group labels indicate sample size and cluster annotation.

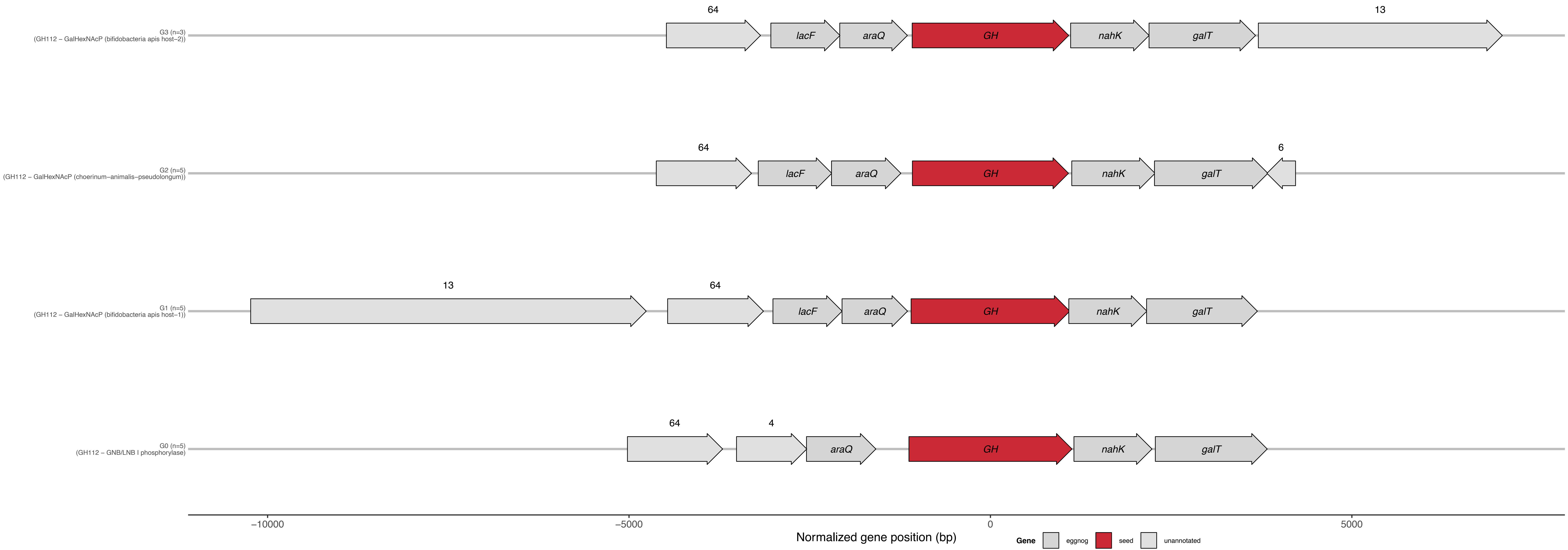

Figure S12. Conserved genomic neighbourhoods of GH112 genes in *Bifidobacterium*. Arrows represent genes colored by annotation source: seed gene (red), reference-matched genes (distinct colors), eggNOG-annotated (dark gray), and unannotated (light gray). Positions are normalized to the seed gene midpoint. Only orthologous clusters present in ≥3 species were retained, with all intervening genes within the outermost shared genes included. Group labels indicate sample size and cluster annotation.

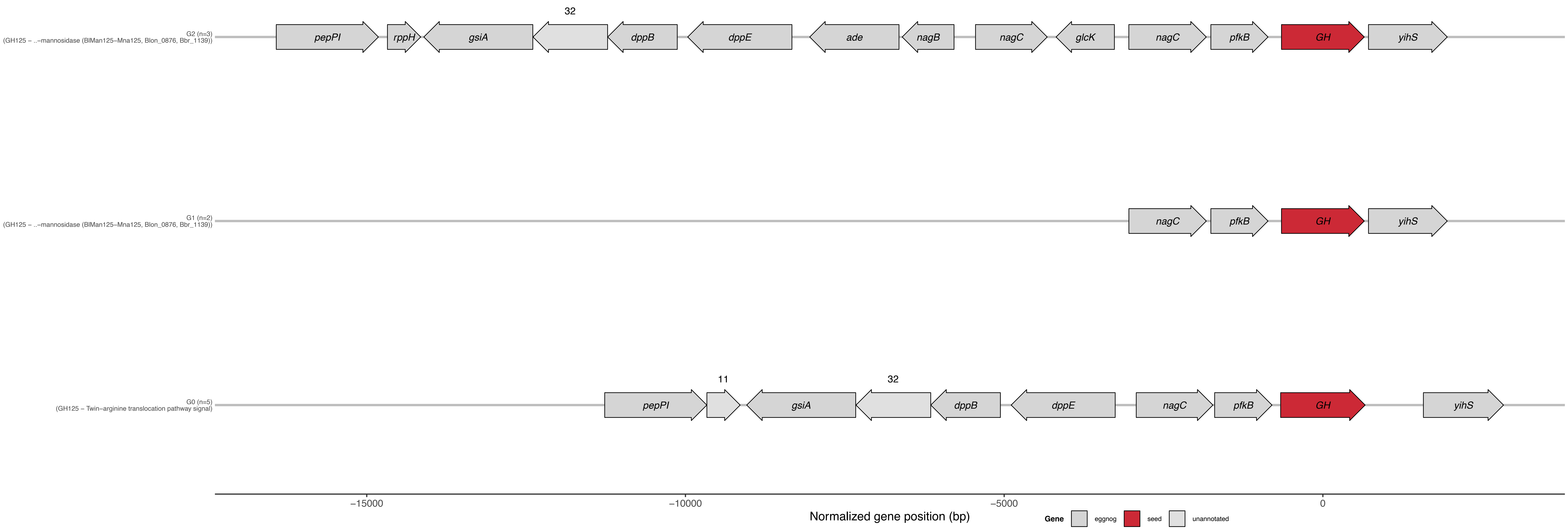

Figure S13. Conserved genomic neighbourhoods of GH125 genes in *Bifidobacterium*. Arrows represent genes colored by annotation source: seed gene (red), reference-matched genes (distinct colors), eggNOG-annotated (dark gray), and unannotated (light gray). Positions are normalized to the seed gene midpoint. Only orthologous clusters present in ≥3 species were retained, with all intervening genes within the outermost shared genes included. Group labels indicate sample size and cluster annotation.

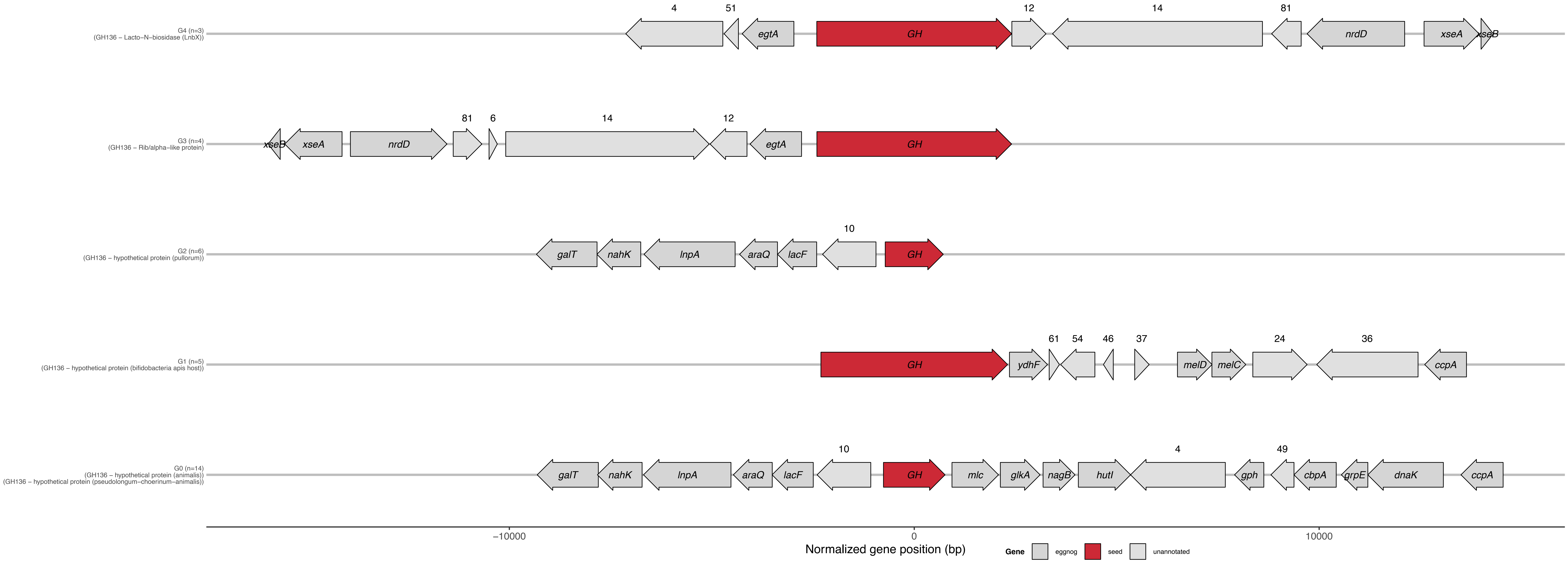

Figure S14. Conserved genomic neighbourhoods of GH136 genes in *Bifidobacterium*. Arrows represent genes colored by annotation source: seed gene (red), reference-matched genes (distinct colors), eggNOG-annotated (dark gray), and unannotated (light gray). Positions are normalized to the seed gene midpoint. Only orthologous clusters present in ≥3 species were retained, with all intervening genes within the outermost shared genes included. Group labels indicate sample size and cluster annotation.

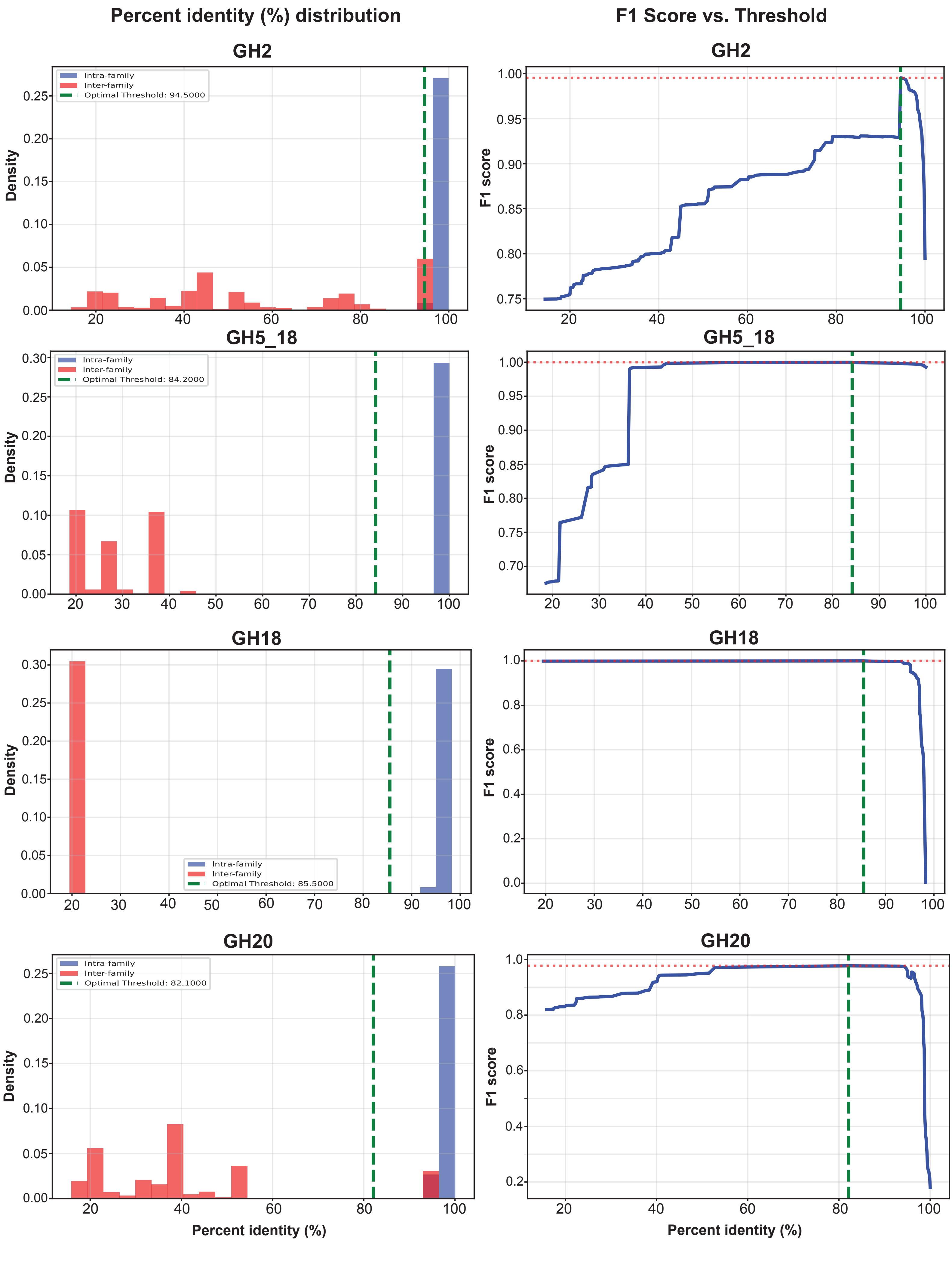

Figure S15. GH-family threshold optimization. Panels on the left-side of the figure shows the distribution of percent identity values for sequence pairs within and between GH families (GH2, GH5_18, GH18 and GH20). Right-side panels shows the corresponding F1 score for threshold-based GH family assignment.

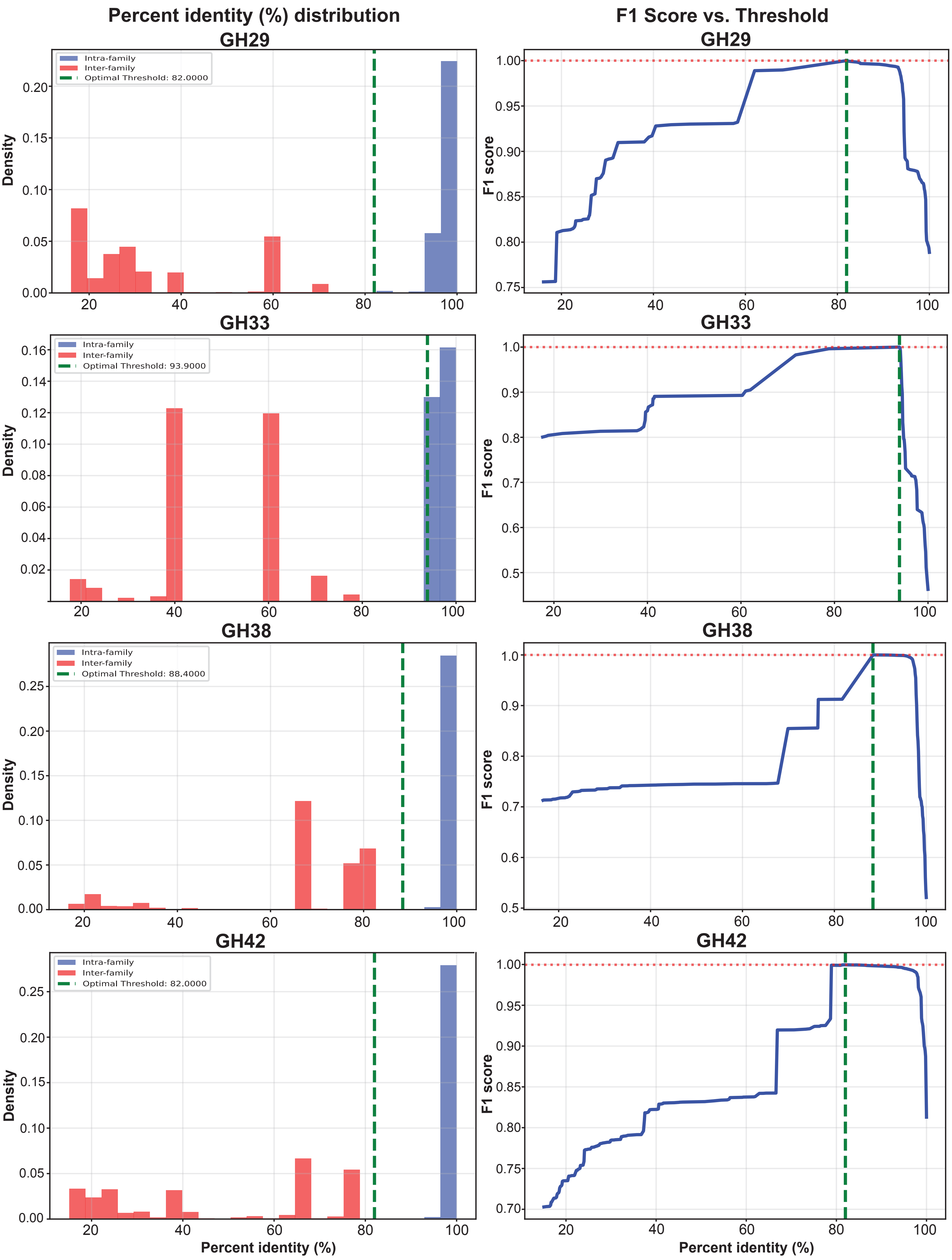

Figure S16. GH-family threshold optimization. Panels on the left-side of the figure shows the distribution of percent identity values for sequence pairs within and between GH families (GH29, GH33, GH38 and GH42). Right-side panels shows the corresponding F1 score for threshold-based GH family assignment.

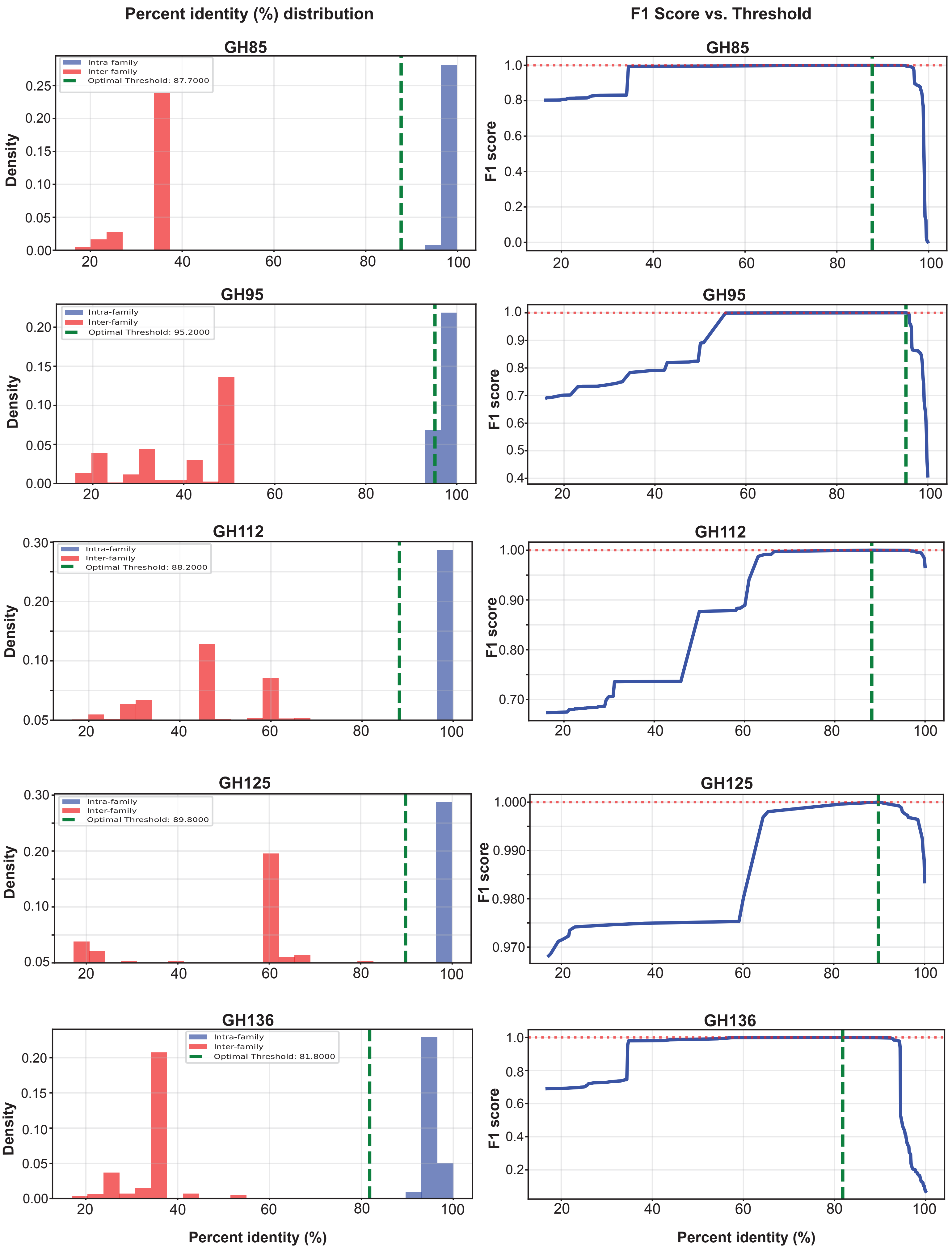

Figure S17. GH-family threshold optimization. Panels on the left-side of the figure shows the distribution of percent identity values for sequence pairs within and between GH families (GH85, GH95, GH112, GH125 & GH136). Right-side panels shows the corresponding F1 score for threshold-based GH family assignment.

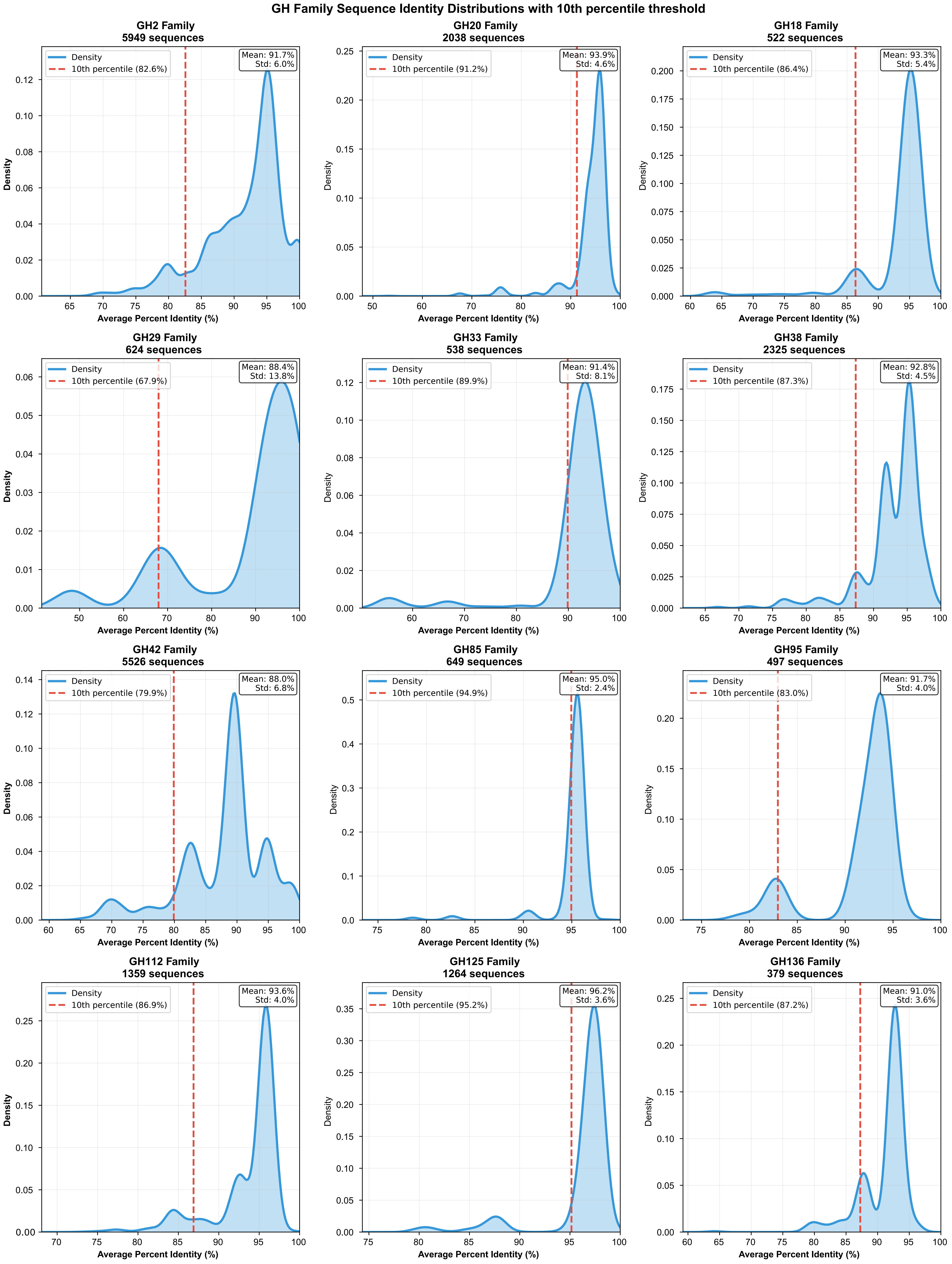

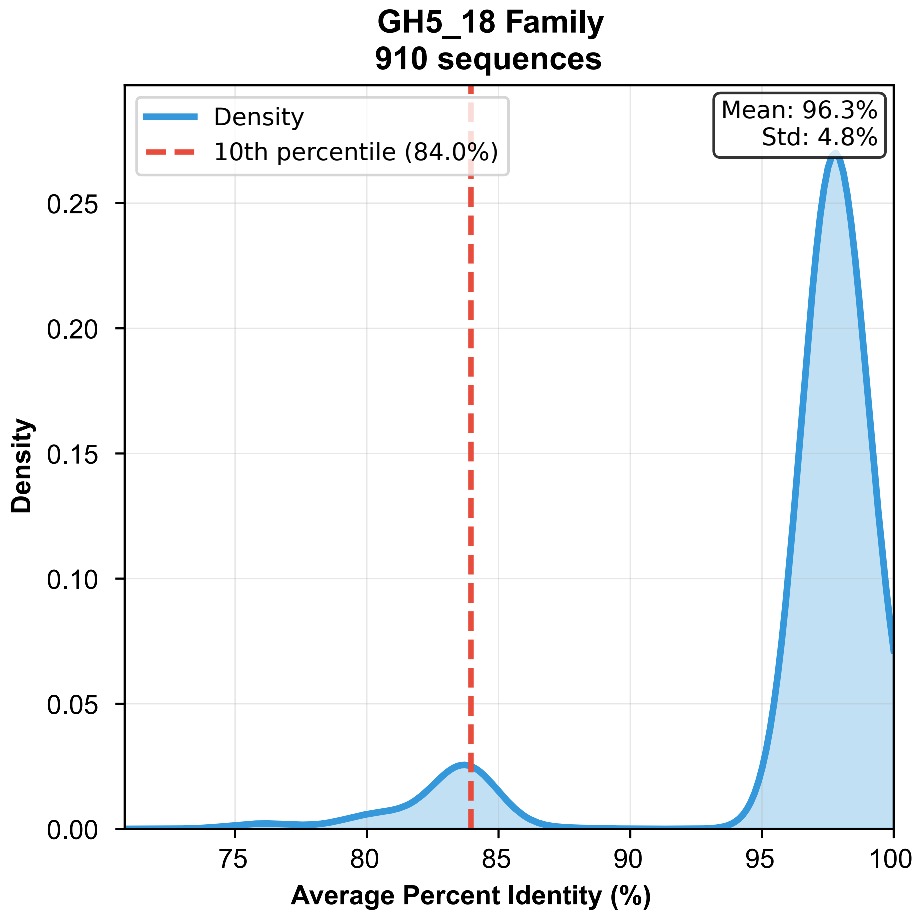

Figure S18. Percentile-based cluster threshold optimization for each GH-family. Density plots of average percent identity for sequences within each glycoside hydrolase (GH) family: GH2, GH5_18, GH18, GH20, GH29, GH33, GH38, GH42, GH85, GH95, GH112, GH125, and GH136. Each panel represents one family, with the number of sequences indicated in the subplot title. The shaded blue curve shows the distribution of intra-cluster average percent identity values, derived from MMseqs2 matches between each sequence and other members of its assigned cluster within the *bifidoAnnotator* reference database (*bifdDB*). This distribution reflects the natural sequence diversity within each GH family's functional clusters and varies considerably across families, motivating the use of family-specific rather than universal thresholds. The dashed red vertical line marks the 10th percentileof this distribution, used as the minimum percent identity cutoff for cluster-level annotation assignment. Mean and standard deviation of percent identity are shown in the upper right of each panel.

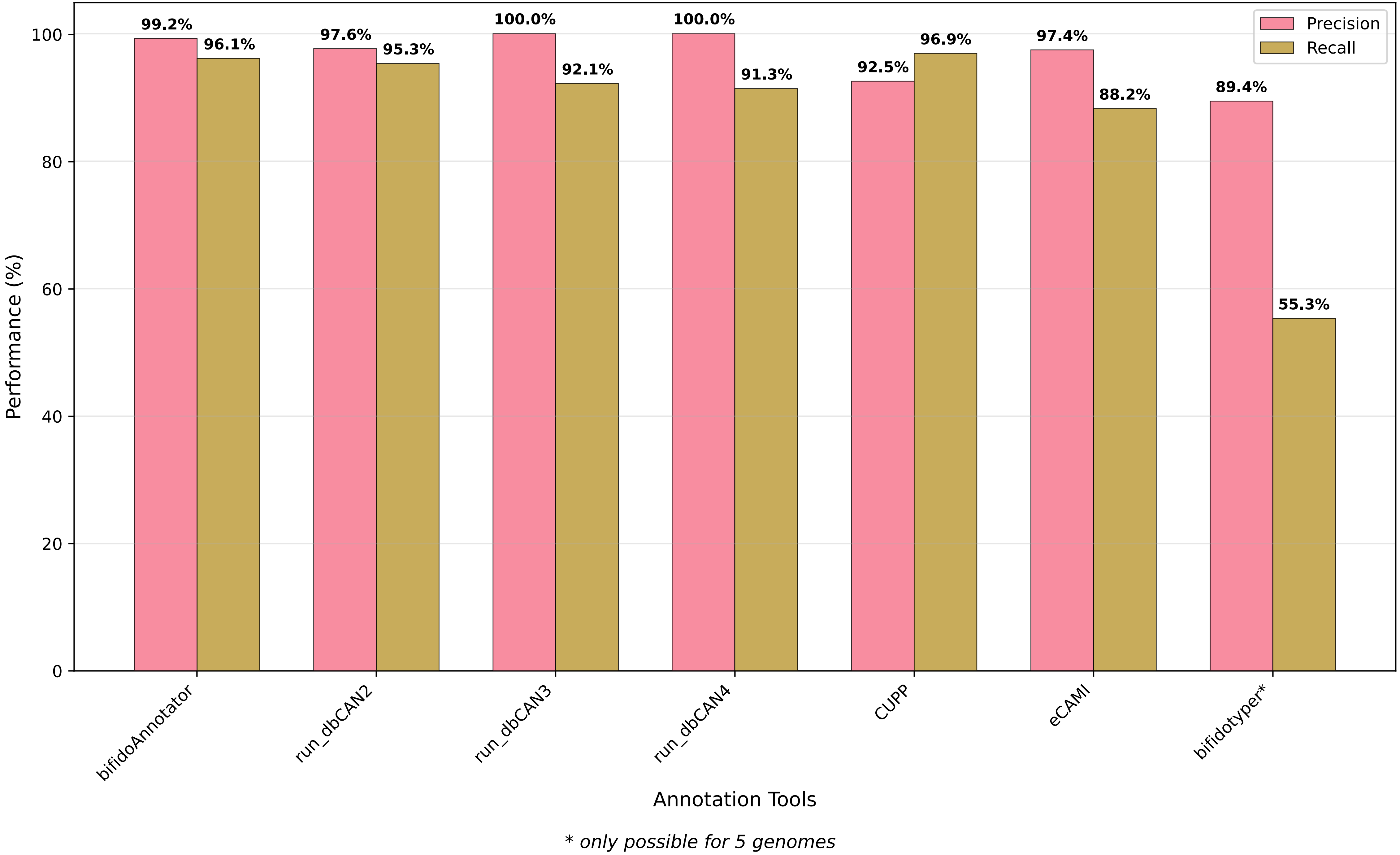

Figure S19. GH-family annotation performance. Precision (dark bars) and recall (light bars) for each annotation tool, ordered by F1-score performance. Tools were benchmarked against CazyDB reference annotations. N.B. bifidotyper performance is based on only 5 genomes.

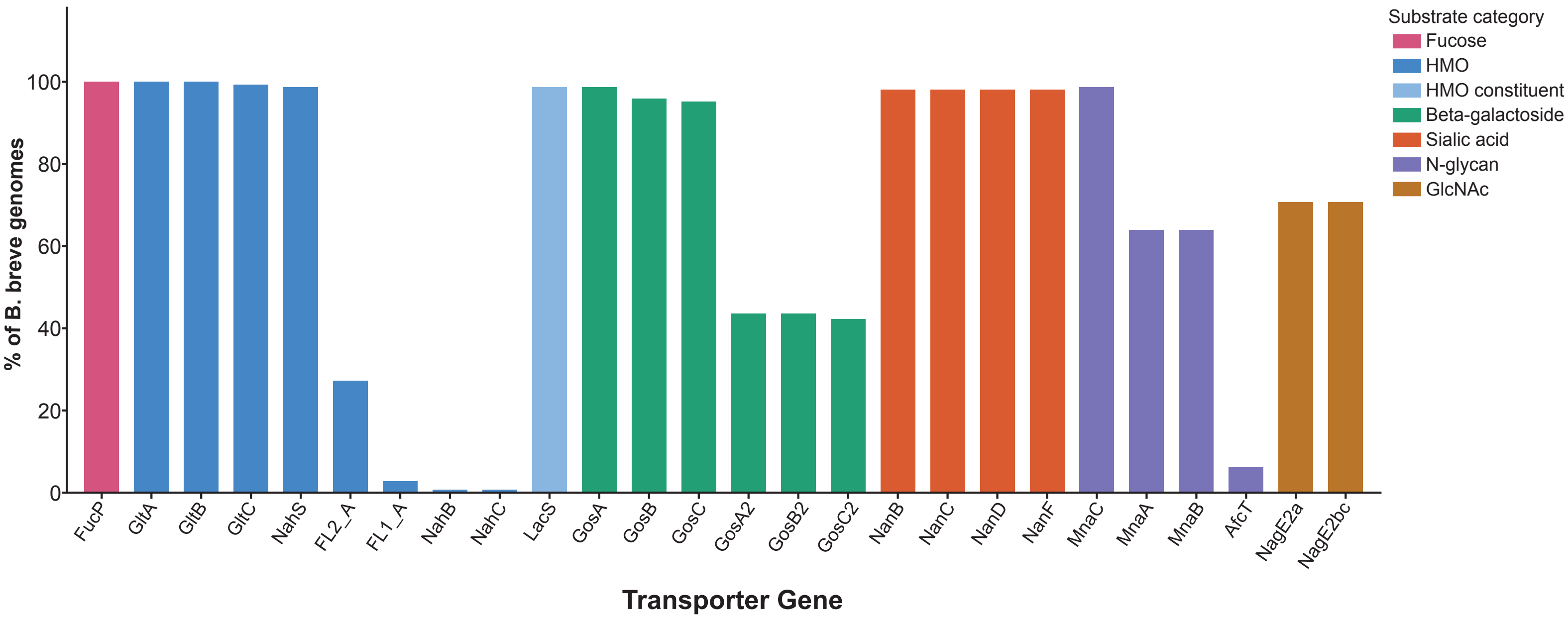

Figure S20. Transporter prevalence across 147 *B. breve* genomes in *bifDB*. Bar plot showing the percentage of *B. breve* genomes carrying each HMG-associated transporter gene annotated by *bifidoAnnotator*. Transporters are grouped and colored by substrate category: fucose (pink), HMO (dark blue), HMO constituent (light blue), beta-galactoside (green), sialic acid (orange), N-glycan (purple), and N-acetylglucosamine (amber).

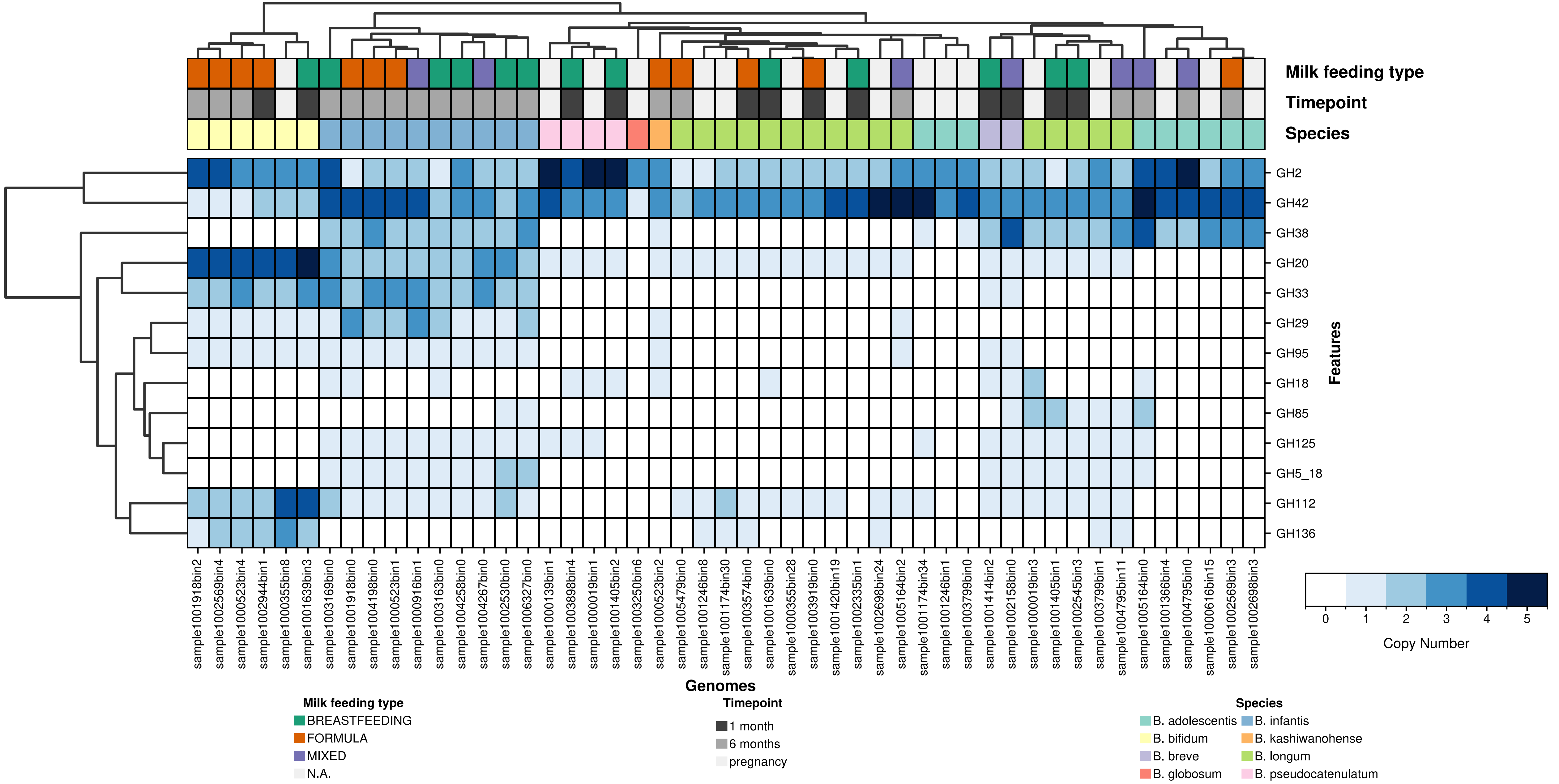

Figure S21. GH family-level copy number heatmap across bifidobacterial MAGs. Heatmap showing GH family copy numbers (white-to-blue scale) across 49 bifidobacterial metagenome-assembled genomes (MAGs) from stool and oral samples of mothers and infants from the Amsterdam Infant Microbiome Study (AIMS). Each row represents one of the 13 HMG-associated GH families; each column represents a genome. Annotation bars indicate species, sampling timepoint and milk feeding type. Genomes and GH families are hierarchically clustered using average linkage with Euclidean distance.

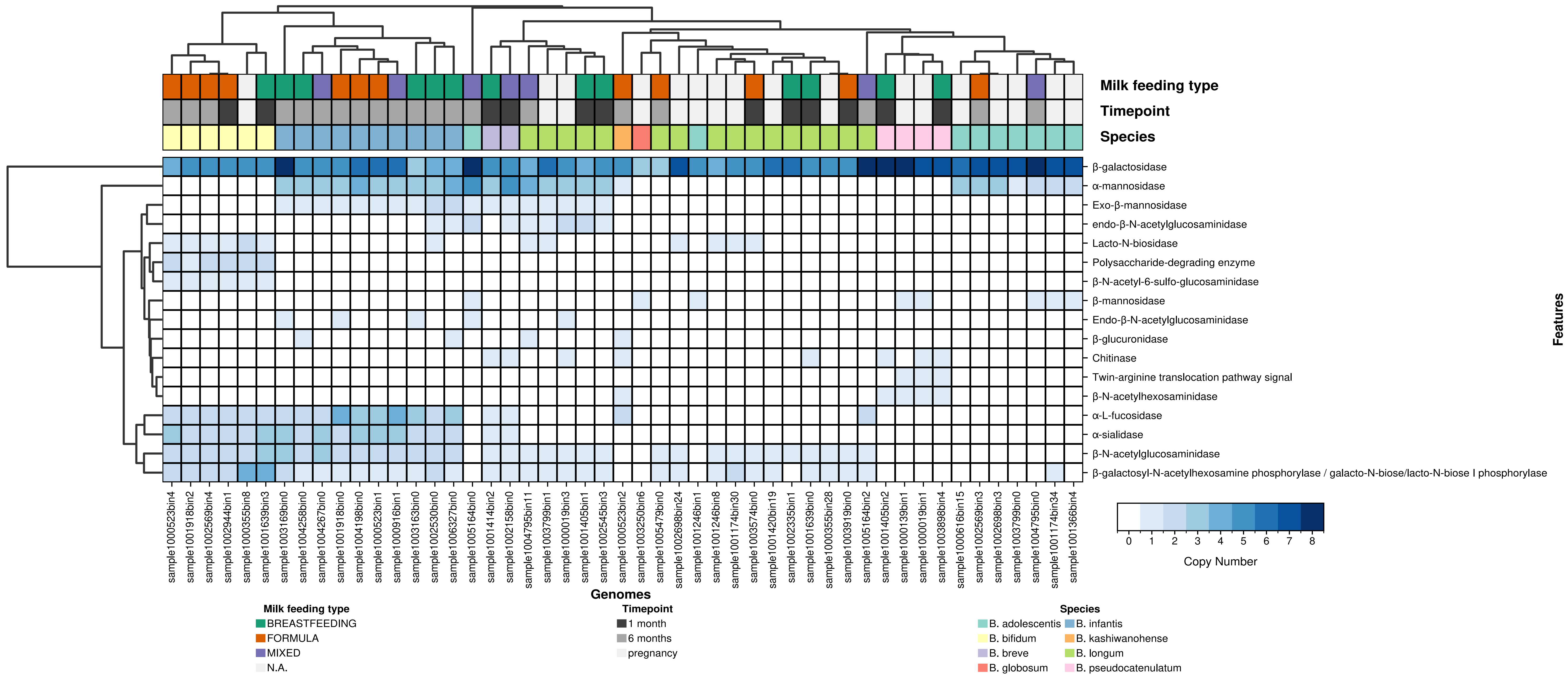

Figure S22. Enzyme function-level copy number heatmap across bifidobacterial MAGs. Heatmap showing enzymatic activity copy numbers (white-to-blue scale) across 49 bifidobacterial metagenome-assembled genomes (MAGs) from stool and oral samples of mothers and infants from the Amsterdam Infant Microbiome Study (AIMS). Each row represents a distinct GH enzymatic activity; each column represents a genome. Annotation bars indicate species, sampling timepoint and milk feeding type. Genomes and enzymatic functions are hierarchically clustered using average linkage with Euclidean distance.

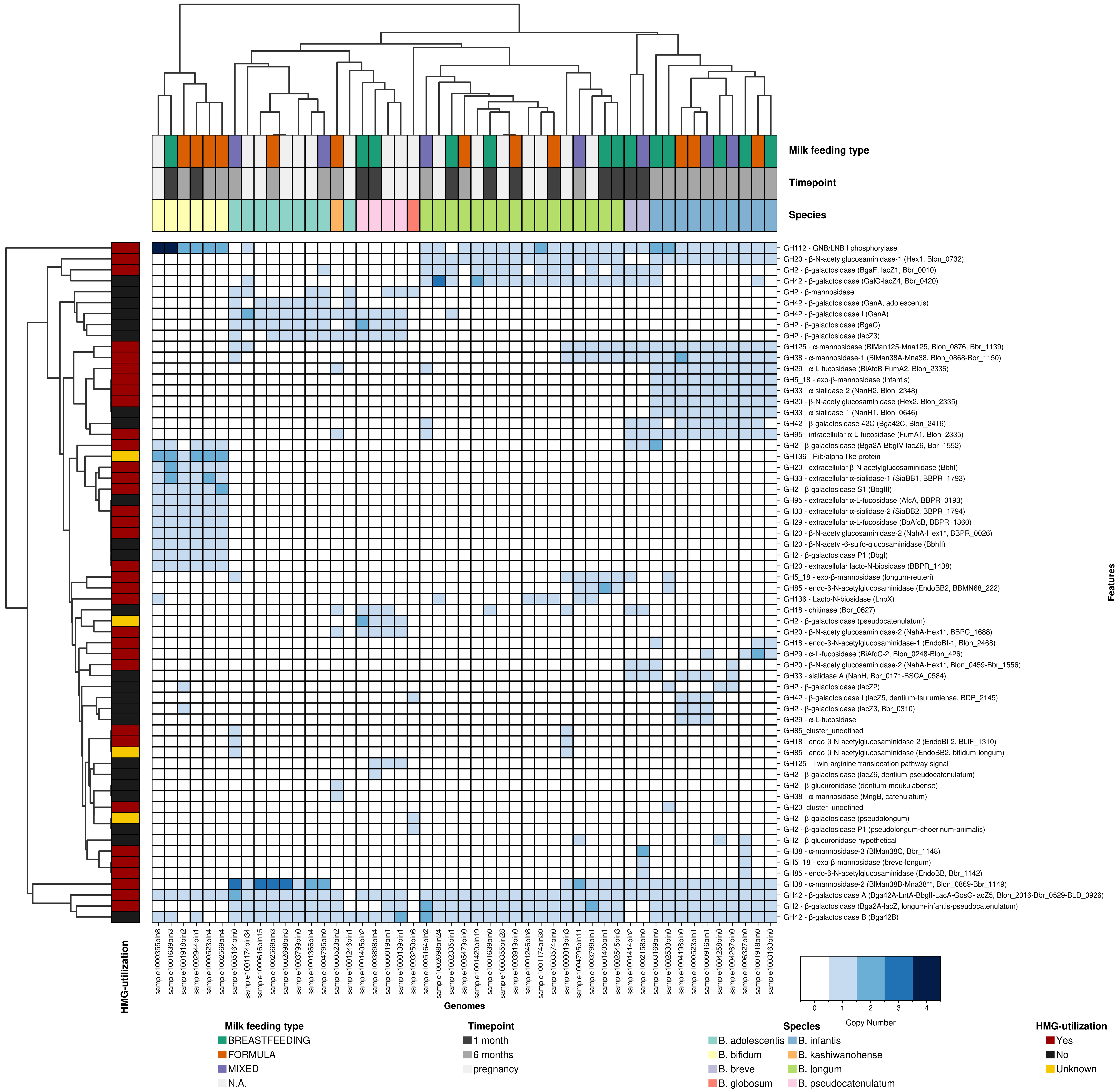

Figure S23. GH cluster-level copy number heatmap across bifidobacterial MAGs. Heatmap showing copy numbers (white-to-blue scale) of 108 fine-grained functional GH clusters (rows) across 49 bifidobacterial MAGs (columns) from the Amsterdam Infant Microbiome Study (AIMS). Annotation bars indicate species, sampling timepoint, milk feeding type, and experimental HMG-utilization status. Genomes and clusters are hierarchically clustered using average linkage with Euclidean distance.

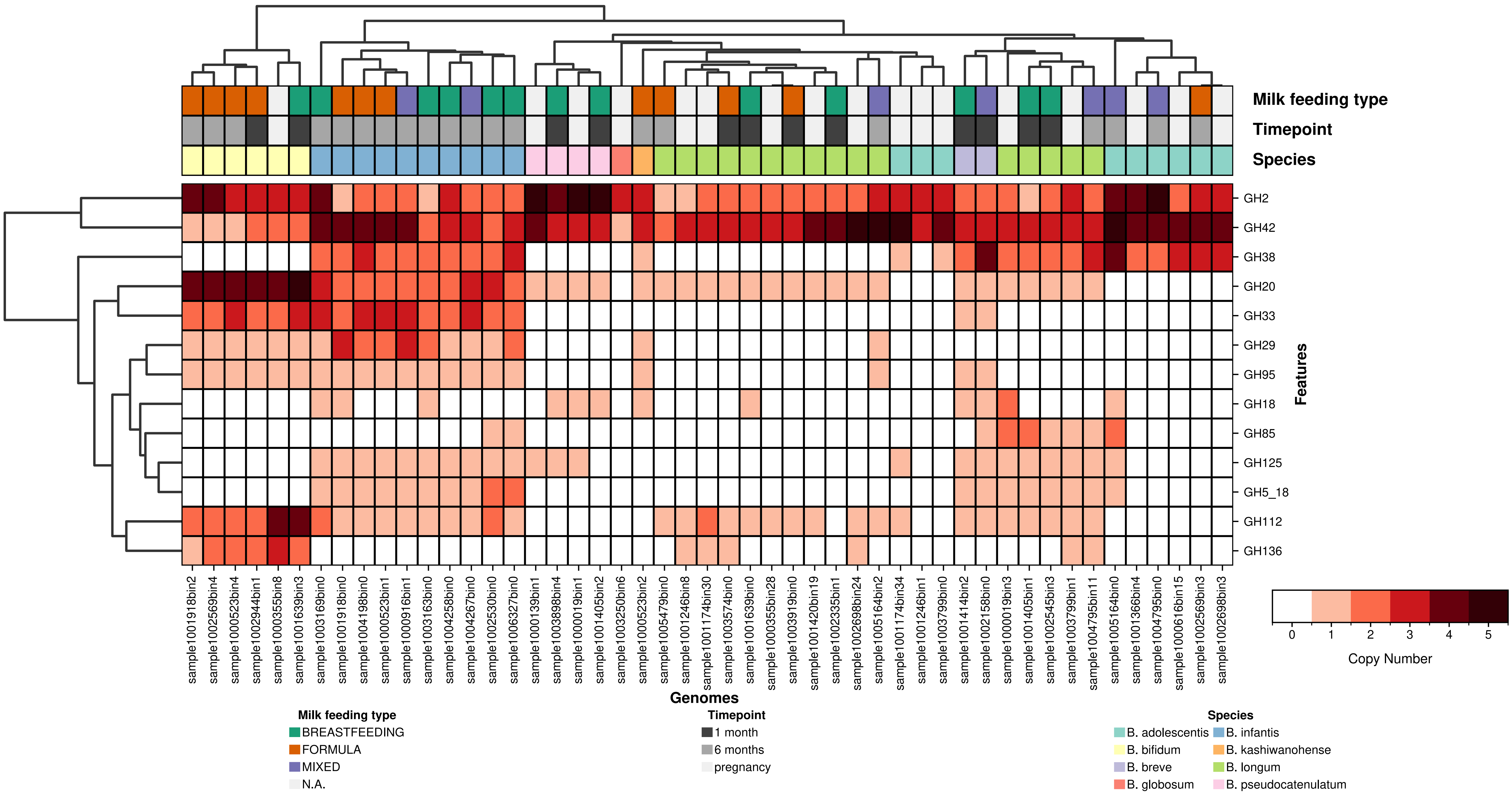

Figure S24. GH family distribution heatmap (red color-scale version). See description Figure S21. Only differences are the color scales used for the heatmap cells (red).

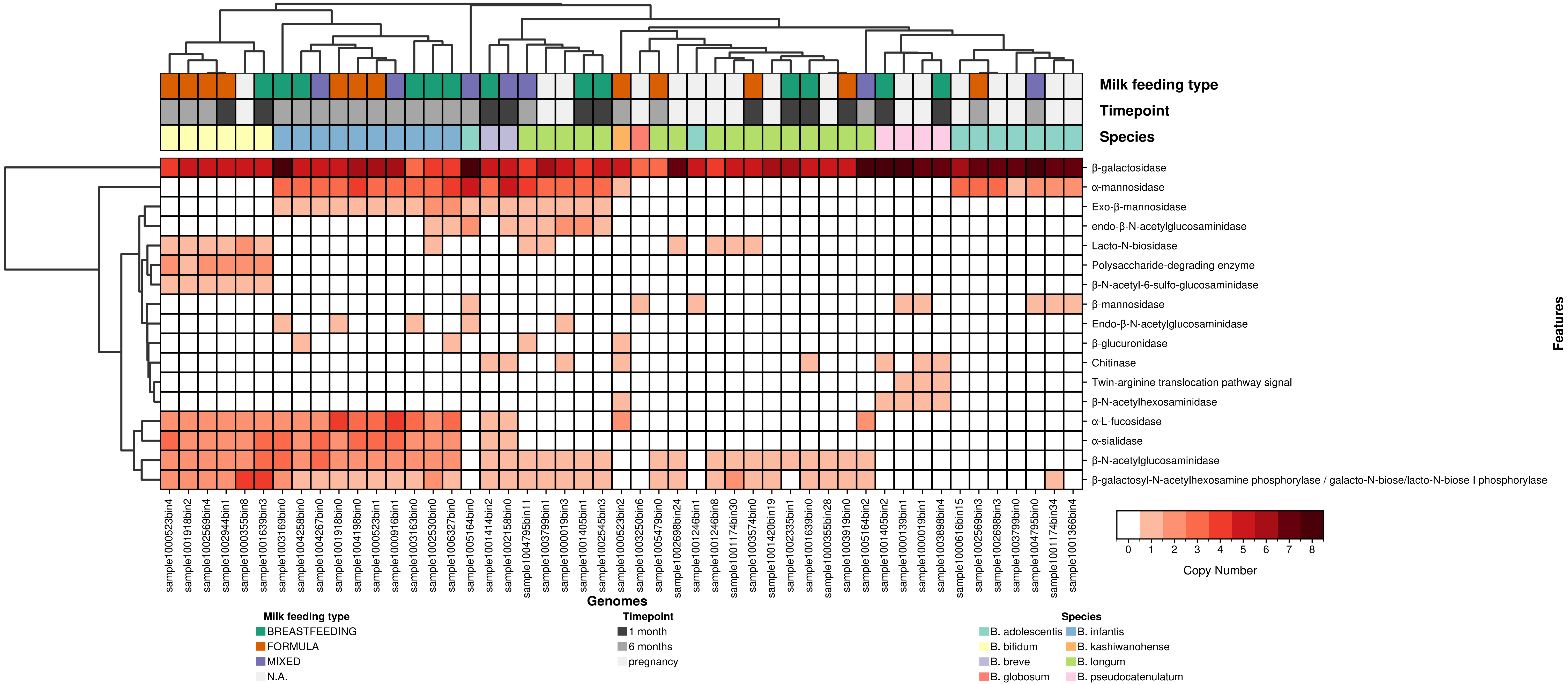

Figure S25. Enzyme function distribution heatmap (red color-scale version). See description Figure S22. Only differences are the color scales used for the heatmap cells (red).

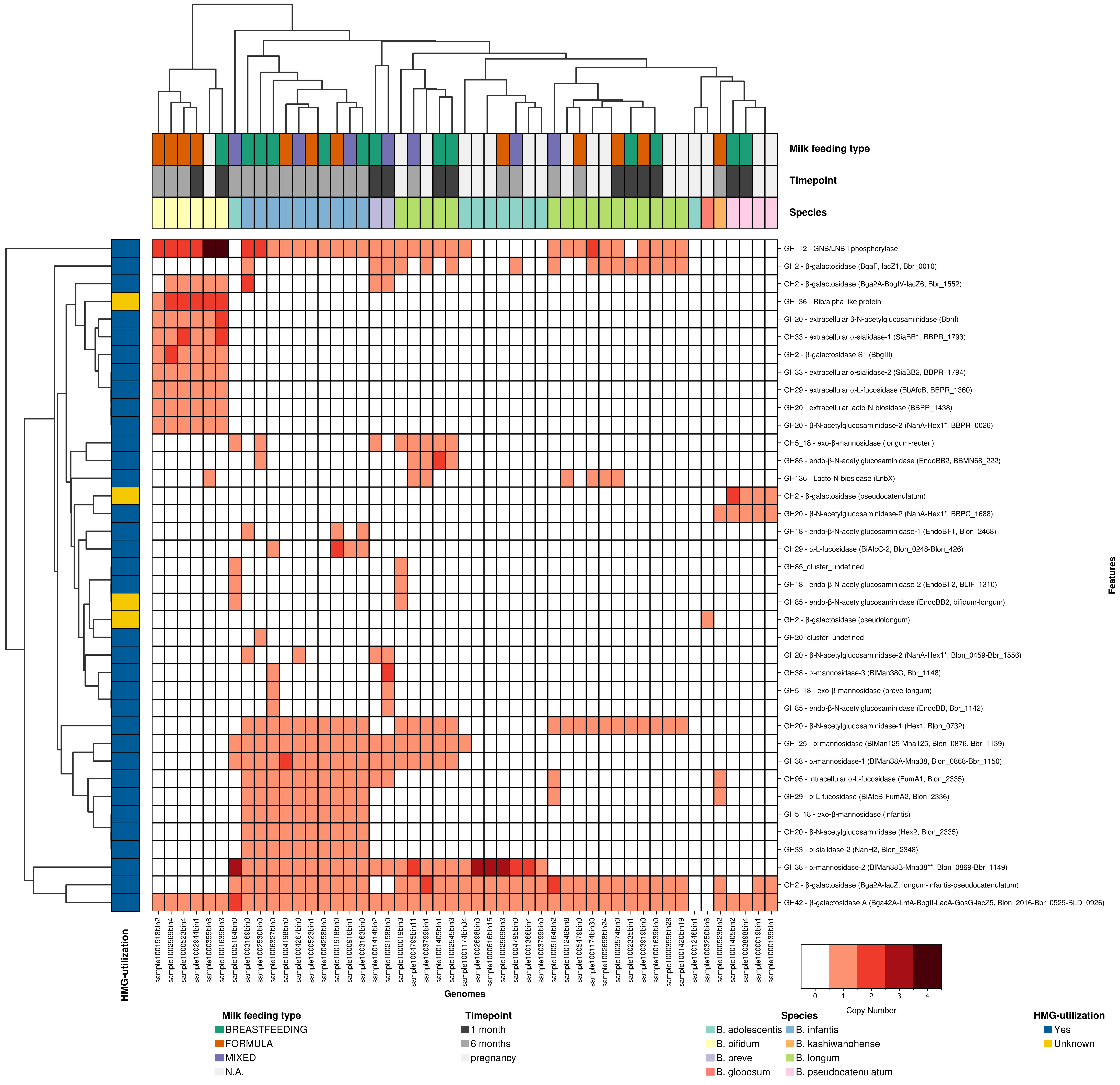

Figure S26. *bifidoAnnotator* homology-based cluster distribution heatmap (red color-scale version). See description Figure S23. Only differences are the color scales used for the heatmap cells (red).
